# Identification of naturally occurring inhibitors in Xian-Ling-Gu-Bao capsule against the glucuronidation of estrogens

**DOI:** 10.3389/fphar.2022.935685

**Published:** 2022-08-04

**Authors:** Liangliang He, Chunxia Xu, Ziying Wang, Shuyi Duan, Jinjin Xu, Chuan Li, Xinsheng Yao, Frank J. Gonzalez, Zifei Qin, Zhihong Yao

**Affiliations:** ^1^ College of Pharmacy, Jinan University, Guangzhou, China; ^2^ School of Chemistry, University of Bristol, Bristol, United Kingdom; ^3^ Department of Pharmacy, The First Affiliated Hospital of Zhengzhou University, Zhengzhou, China; ^4^ State key Laboratory of Drug Research, Shanghai Institute of Materia Medica, Chinese Academy of Sciences, Shanghai, China; ^5^ International Cooperative Laboratory of Traditional Chinese Medicine Modernization and Innovative Drug Development Ministry of P.R. China, Jinan University, Guangzhou, China; ^6^ Laboratory of Metabolism, Center for Cancer Research, National Cancer Institute, National Institutes of Health, Bethesda, MD, United States

**Keywords:** Xian-Ling-Gu-Bao capsule, estrogens, glucuronidation, inhibitory effects, UDP-glucuronosyltransferase

## Abstract

Xian-Ling-Gu-Bao (XLGB) capsule, a well-known traditional Chinese medicine prescription, is widely used for the treatment of osteoporosis. It could significantly increase the levels of estrogen in ovariectomized rats and mice. However, this working mechanism has not been well elucidated. Considering that UDP-glucuronosyltransferase (UGT) enzymes are the important enzymes that inactivate and regulate estrogen activity *in vivo*, this study aimed to identify the bioactive compounds from XLGB against the glucuronidation of estrogens. First, thirty compounds were considered as candidate bioactive compounds based on our previous studies including pharmacological evaluation, chemical profiles, and metabolic profiles. Second, the characteristics of estrogen glucuronidation by uridine diphosphate glucuronic acid (UDPGA)-supplemented human liver microsomes (HLM), human intestine microsomes (HIM), and expressed UGT enzymes were determined, and the incubation systems of their key UGT enzymes were optimized. Then, inhibitory effects and mechanisms of XLGB and its main compounds toward the key UGT isozymes were further investigated. As a result, estrogen underwent efficient glucuronidation by HLM and HIM. UGT1A10, 1A1, and 2B7 were mainly responsible for the glucuronidation of estrone, β-estradiol, and estriol, respectively. For E1 and E2, UGT1A10 and 1A1 tended to mediate estrogen-3-*O*-glucuronidation, while UGT2B7 preferred catalyzing estrogen-16-*O*-glucuronidation. Furthermore, the incubation system for active UGT isoforms was optimized including Tris-HCl buffer, detergents, MgCl_2_ concentration, β-glucuronidase inhibitors, UDPGA concentration, protein concentration, and incubation time. Based on optimal incubation conditions, eleven, nine, and nine compounds were identified as the potent inhibitors for UGT1A10, 1A1, and 2B7, respectively (IC_50_ < 4.97 μM and K_i_ < 3.35 μM). Among them, six compounds (bavachin, isobavachin, isobavachalcone, neobavaisoflavone, corylifol A, and icariside II) simultaneously demonstrated potent inhibitory effects against these three active enzymes. Prenylated flavanols from *Epimedium brevicornu* Maxim., prenylated flavonoids from *Psoralea corylifolia* L., and salvianolic acids from *Salvia miltiorrhiza* Bge. were characterized as the most important and effective compounds. The identification of potent natural inhibitors of XLGB against the glucuronidation of estrogen laid an important foundation for the pharmacodynamic material basis.

## Graphical Abstract

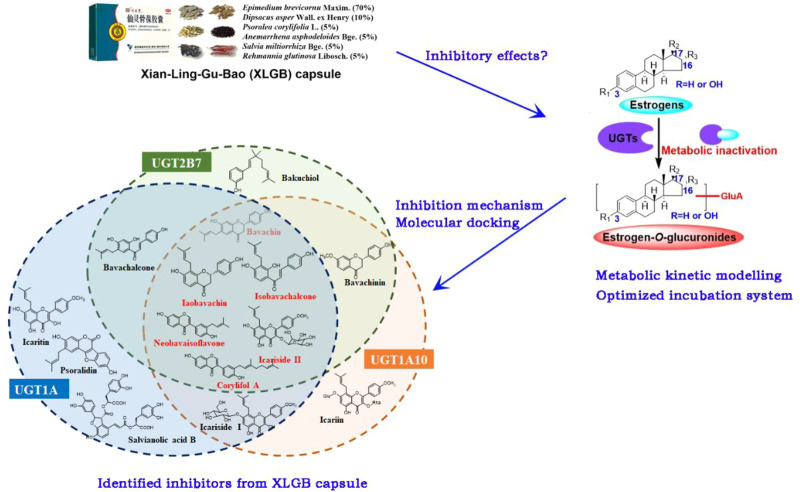

## Introduction

Among the sex steroid hormones, estrogens (estrone, estradiol, estriol, etc.) distinguish themselves for the variety of their target tissues. Indeed, estrogen receptors are present in most cells in mammals, thus enabling these hormones to regulate or interfere with many metabolic pathways, especially in maintaining bone homeostasis and regulating remodeling (Maggi and Della Torre, 2018). The imbalance of estrogens often leads to the disorder of physiological function and the occurrence of various diseases (Manolagas et al., 2013). For instance, estrogen depletion is an important factor for osteoporosis in postmenopausal women (Manolagas et al., 2013). At present, estrogen supplements are widely used to prevent and treat osteoporosis because they could reduce bone loss in postmenopausal women (Manolagas et al., 2013). Currently, it is encouraging to develop alternative agents from herbal products with far fewer side effects for the prevention and treatment of osteoporosis.

Xian-Ling-Gu-Bao (XLGB) capsule consists of six commonly used herbs: Epimedii Folium (the dried leaves of *Epimedium brevicornu* Maxim.) (70%), Dipsaci Radix (the dried roots of *Dipsacus asper* Wall. ex Henry) (10%), Psoraleae Fructus (the dried seeds of *Psoralea corylifolia* L.) (5%), Anemarrahenae Rhizoma (the dried rhizomes of *Anemarrhena asphodeloides* Bge.) (5%), Salviae Miltiorrhizae Radix et Rhizoma (the dried roots and rhizomes of *Salvia miltiorrhiza* Bge.) (5%), and Rehmanniae Radix (the dried roots of *Rehmannia glutinosa* Libosch.) (5%) (Chen et al., 2019). Its abundant compounds (e.g., icariin and icaritin) exhibited a high affinity for estrogen receptors (Mok et al., 2010) and a significant estrogen-like effect on the promotion of estrogens (Qin et al., 2005; Xin et al., 2010). Its safety and effectiveness for the treatment of osteoporosis and osteonecrosis in postmenopausal women have been proven by multicenter, randomized, double-blind, placebo-controlled clinical trials (Zhu et al., 2012; Li et al., 2018; Chen et al., 2019). Remarkable clinical efficacy attracts increasing interest in the fields of herbal compounds, quality control, metabolism, and pharmacological research studies.

Prenylated flavonoid glycosides (or flavone, isoflavones, and chalcones), saponins, and coumarins were likely the main active compounds responsible for the therapeutic effect (Guan et al., 2011; Dai et al., 2013). Their respective content levels in XLGB were also performed (Yao et al., 2017a). Metabolism and pharmacokinetics research studies also demonstrated that prenylated flavonoids, coumarins, and saponins were the most abundant xenobiotics in rats (Yao et al., 2017b; Tang et al., 2020), while hydrolysis, hydroxylation, glucuronidation, and sulfation were the major metabolic pathways (Geng et al., 2014). These also benefit our understanding of the pharmacodynamic material basis responsible for the therapeutic effects of XLGB.

The clinical investigation of XLGB for the treatment of menopausal syndrome found that it could significantly improve the clinical symptom and raise the level of serous estradiol while lowering the risk of endometrial hyperplasia (Lou et al., 2009). In addition, XLGB has proved to have preventive effects on ovariectomized (OVX)-induced bone loss in mice (Wang et al., 2015b) and in rats (Chen et al., 2016). Also, it could significantly increase the levels of estrogens (Tang et al., 2021). Furthermore, XLGB was usually used as a positive control drug for the evaluation of anti-osteoporotic activity (Li et al., 2013). In addition, it should be noted that the use of XLGB has been approved by the China Food and Drug Administration for the treatment of osteoporosis, osteoarthritis, aseptic osteonecrosis, and fractures (Chen and O'Keefe, 2018).

**FIGURE 1:**
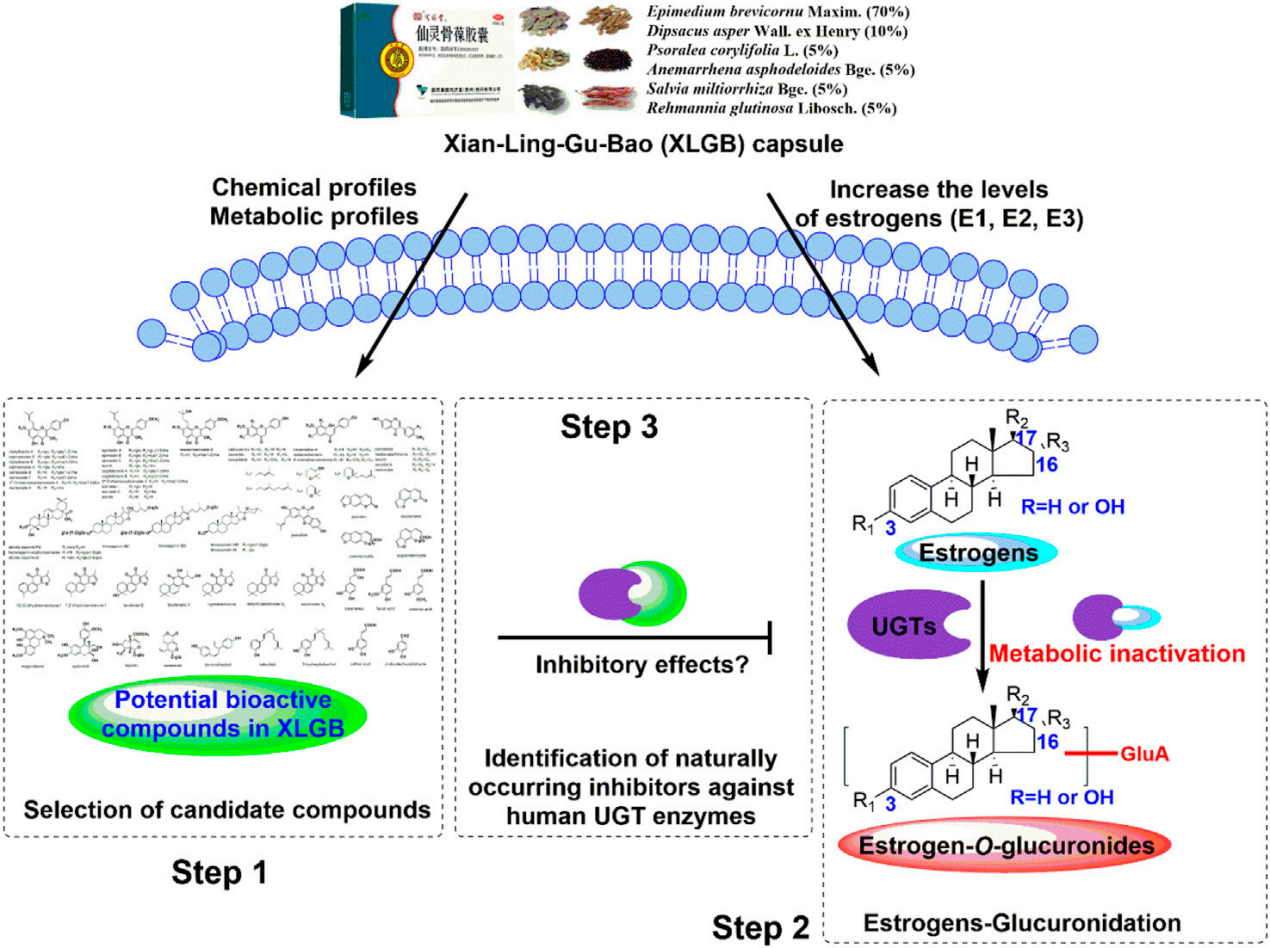
Analytical strategy for the identification of naturally occurring inhibitors against the glucuronidation of estrogens.

However, estrogens were subjected to undergo massive glucuronidation by UDP-glucuronosyltransferases (UGTs), and the conjugated glucuronides were inactive products for estrogens (Sneitz et al., 2013). Therefore, UGT enzymes are the important enzymes that inactivate and regulate the *in vivo* levels of estrogen (Raftogianis et al., 2000; Guillemette et al., 2004; Lindsey, 2014). So far, whether the *in vivo* xenobiotics of XLGB could increase estrogen levels by inhibiting the function of active UGT enzymes that could catalyze the glucuronidation of estrogens remains unclear. For this goal, we first determine the selection of candidate compounds based on the chemical profiles and metabolic profiles of XLGB. In addition, the main UGT isozymes for estrogen glucuronidation were identified, and the corresponding incubation conditions were also optimized. Furthermore, the inhibitory effects and mechanisms of XLGB-related xenobiotics against these active UGT enzymes were elucidated (Figure 1). This study would provide a solid basis for pharmacodynamic substances of XLGB based on the metabolic regulation of estrogens.

## Materials and methods

### Chemicals and reagents

Estrone (E_1_), β-estradiol (E_2_), estriol (E_3_), estrone 3-*O*-glucuronide sodium salt (E_1_-3-*O*-G), *β*-estradiol 3-*O*-glucuronide sodium salt (E_2_-3-*O*-G), estriol 3-*O*-glucuronide sodium salt (E_3_-3-*O*-G), 4-methylumbelliferone (4-MU), 4-methylumbelliferone glucuronide (4-MU-G), zidovudine (AZT), atazanavir, diclofenac, uridine diphosphate glucuronic acid (UDPGA), D-saccharic-1,4-lactone saccharolactone, magnesium chloride (MgCl_2_), and alamethicin were purchased from Sigma-Aldrich (St. Louis, MO). *β*-estradiol 17-*O*-glucuronide sodium salt (E_2_-17-*O*-G), estriol 16*α*-*O*-glucuronide sodium salt (E_3_-16-*O*-G), and zidovudine-glucuronide (AZT-G) were purchased from Toronto Research Chemicals (Toronto, ON, Canada). Four detergents, including Brij35, Brij58, CHAPS, and Triton X-100, and multiple components of XLGB, including icariin, icariside I, icariside II, icaritin, cycloicaritin, epimedin A, epimedin B, epimedin C, magnoflorine, bavachin, isobavachin, bavachalcone, isobavachalcone, neobavaisoflavone, bavachinin, corylifol A, corylin, psoralidin, psoralen, isopsoralen, bakuchiol, asperosaponin VI, sweroside, loganin, salvianolic acid B, tanshinone IIA, 15,16-dihydrotanshinone I, timosaponin B-II, and catalpol, were purchased from Shanghai Winherb Medical Technology Co., Ltd. (Shanghai, China). The purity of each compound mentioned earlier was more than 98%, and other reagents were of analytical grade or better.

Pooled human liver microsomes (HLM), pooled human intestine microsomes (HIM), and thirteen expressed human UGT isoforms (UGT1A1, 1A3, 1A4, 1A6, 1A7, 1A8, 1A9, 1A10, 2B4, 2B7, 2B10, 2B15, and 2B17) were all obtained from Corning Biosciences (New York, United States).

### Analytical conditions

LC-MS/MS analysis was performed to quantify three endogenous estrogens (E_1_, E_2,_ and E_3_) and their generated glucuronides by Xevo TQ-XS (Waters Corporation, Milford, United States). UHPLC separation was carried out using an ACQUITY HSS T3 column (1 mm × 50 mm, 1.8 µm) at a flow rate of 0.4 ml/min, and the mobile phase consisted of water (A) and acetonitrile (B) (both containing 0.1% formic acid). The gradient elution programs for E_2_ and its glucuronides were as follows: 10–20% B at 0–2 min, 20–40% B at 2–3 min, 40–50% B at 3–4 min, 40–50% B at 4–4.5 min, 50–90% B at 4.5–5 min, 90–100% B at 5–7 min, maintained 100% B at 7–7.1 min, and 100%–10% B at 7.1–8 min. The gradient systems for E_1_, E_3,_ and their glucuronides were as follows: 2%–10% B at 0–2 min, 10%–40% B at 2–3 min, 40–50% B at 3–5 min, 50–90% B at 5–6 min, 90–100% B at 6–7 min, maintained 100% B at 7–8 min, and 100–2% B at 8–9 min. The multiple reaction monitoring (MRM) mode was operated in positive ion mode. The detailed parameters were set as follows: source temperature 150°C, desolvation temperature 550°C, capillary voltage 3.3 kV, desolvation gas 1000 L/h, cone gas 150 L/h, the *m/z* 271.17 → 253.04 transition with a collision energy (CE) of 10 eV for E_1_, E_3_, and their glucuronides, and the *m/z* 273.18 → 106.91 transition with a CE of 25 eV for E_2_ and its glucuronides. All experimental data were collected in centroid mode and analyzed using MassLynx 4.1 software (Waters Corporation, Milford, MA, United States).

In addition, the separation of 4-MU, AZT, and their generated glucuronides was performed by an ACQUITY UHPLC I-Class system equipped with a PDA detector (Waters Corporation, Manchester, United Kingdom). Chromatographic separation was achieved with a BEH C_18_ column (2.1 mm × 50 mm, 1.7 μm). The mobile phase also consisted of water (A) and acetonitrile (B) (both containing 0.1% formic acid). The flow rate was 0.4 ml/min, and the detection wavelength was 316 and 267 nm for 4-MU and AZT, respectively; 4-MU and 4-MU-G were eluted using the following gradient: 5–60% B at 0–3.5 min, 60–65% B at 3.5–3.8 min, 65–100% B at 3.8–4 min, maintained 100% B at 4–5 min, and 100–5% B at 5–6 min. Also, the gradient program for AZT and AZT-G was as follows: maintained 2% B at 0–1 min, 2–5% B at 1–1.5 min, 5–40% B at 1.5–3.5 min, maintained 40% B at 3.5–4 min, 40–100% B at 4–4.5 min, maintained 100% B at 4.5–5 min, and 100–2% B at 5–6 min.

### Glucuronidation assay

The glucuronidation assay was conducted as described previously (Qin et al., 2018; Xu et al., 2018). In brief, incubation mixtures (100 μL) contained Tris-HCl buffer solution (50 mM, pH = 7.4), microsomes (HLM, HIM, 0.5 mg/ml) or UGT enzymes (1 mg/ml), MgCl_2_ (0.88 mM), alamethicin (22 μg/ml), and D-saccharic-1,4-lactone (4.4 mM) and respective substrate. The volume of organic solvent in incubation mixtures was kept below 1%. After pre-incubation at 37°C for 5 min, the reaction was commenced with the addition of UDPGA (4 mM) and subsequently incubated at 37°C for a period. The reaction was terminated by adding 100 μL of cold acetonitrile. Then, the incubation mixtures were vortexed and centrifuged at 13,800 g for 10 min to collect the supernatant for analysis.

### Enzyme kinetic evaluation

Thirteen recombinant UGT isoforms (UGT1A1, 1A3, 1A4, 1A6, 1A7, 1A8, 1A9, 1A10, 2B4, 2B7, 2B10, 2B15, and 2B17) were incubated with E_1_, E_2_, and E_3_ (2 and 10 μM) to identify the most important contributors, respectively. A series of E_1_ solutions (0.625–80 µM) were separately incubated in HLM, HIM, and UGT1A3 for 90 min. In addition, serial E_2_ solutions (0.5–150 µM) were incubated in HLM, HIM, UGT1A1, and UGT1A10 for 60 min, respectively, while E_3_ solutions (0.5–150 µM) were incubated in HLM, HIM, and UGT2B7 for 30 min, respectively. All experiments were performed in triplicate.

Glucuronidation rates were expressed as the amounts of formed metabolites per minute per milligram protein (pmol/min/mg protein) based on Eq. 1. Furthermore, kinetic characteristics were evaluated from the suitable curves based on the profile of the Eadie–Hofstee plot, and corresponding kinetic parameters were estimated using Eqs 2–4. Appropriated model fitting was performed by GraphPad Prism V5 software (San Diego, CA).

The parameter *K*_*m*_ is the Michaelis–Menten constant; *V*_*g*_ is the formation rate of metabolites; *V*_*max*_ is the maximal rate of metabolites; *K*_*si*_ is the substrate inhibition constant; *S*_*50*_ is the substrate concentration resulting in 50% of *V*_*max*_. *n* is the Hill coefficient. The intrinsic clearance (*CL*_*int*_) was derived from *V*_*max*_ */K*_*m*_ for Michaelis–Menten and substrate inhibition models, and the maximal clearance (*CL*_*max*_) was obtained using Eq. 5 for Hill kinetics.

$$V_{g}=\frac{C_{s}*V}{C_{protein}*T*V_{protein}}.$$ (1)

$$V=\frac{Vmax\times \left[S\right]}{Km+\left[S\right]},$$ (2)

$$V=\frac{V\max\times \left[S\right]}{Km+\left[S\right]\left(1+\frac{\left[S\right]}{Ksi}\right)},$$ (3)

$$V=\frac{Vmax\times {\left[S\right]}^{n}}{S_{50}^{n}\times {\left[S\right]}^{n}},$$ (4)

$$CL_{max}=\frac{V_{max}}{S_{50}}\times \frac{n-1}{n{\left(n-1\right)}^{1/n}}.$$ (5)

### Optimization of UGT1A10, 1A1, and 2B7 incubation systems

β-estradiol (E2) and zidovudine (AZT) were the recognized probe substrates for UGT1A1 and UGT2B7, respectively (Lv et al., 2019; Gao et al., 2020). However, there is no specific substrate of UGT1A10 so far. Traditionally, 4-methylumbelliferone (4-MU) was used as the substrate of UGT1A10 with K_m_ values of about 60 μM as described previously (Xin et al., 2016; Gao et al., 2020). E_2_ (10 μM for UGT1A1), 4-MU (60 μM for UGT1A10), and AZT (800 μM for UGT2B7) were used as the substrates to evaluate their respective metabolic activities. The substrate concentration was close to or below the corresponding *K*_*m*_ reported previously (Wang et al., 2015a; Xing et al., 2020). Different buffer concentrations (12.5–100 mM) and pH values (6.5–9.0) were used to evaluate their respective glucuronidation activities. Other conditions were identical to those used in the glucuronidation assay.

In addition, the effects of alamethicin (20 μg/ml) and four detergents (Brij35, Brij58, Triton X-100, and CHAPS; 100 μg/ml) for glucuronidation were estimated; 4-MU and AZT were both conducted in 100 mM Tris buffer (pH 7.4), while E_2_ was incubated in 50 mM Tris buffer (pH 8.0). Furthermore, MgCl_2_ and UDPGA were also evaluated with final concentrations ranging from 0 to 10 mM and from 0 to 8 mM, respectively. To investigate the effects of β-glucuronidases, 5 mM saccharolactone (a β-glucuronidase inhibitor) was added to the incubation mixtures.

Moreover, for time-dependent assay, the reaction system was expanded to a total volume of 1 ml under the above-optimized conditions. After initiating the reaction, a subsample (100 μL) of the incubation mixtures was immediately removed and terminated by cold acetonitrile (100 μL) at eight time points (15, 30, 45, 60, 90, 120, 150, and 180 min). For the protein concentration-dependent assay, various concentrations of UGT1A10, UGT1A1, or UGT2B7 (12.5, 25, 50, 100, and 250 μg/ml) were added to the incubation mixtures.

### Inhibitory effects of natural components in XLGB against UGT enzymes

The inhibitory effects of natural components in XLGB toward human UGT1A10, 1A1, and 2B7 were determined in the absence and presence of these compounds at four concentrations (0, 1, 10, and 100 µM) based on optimized incubation conditions. The final volume of solvent in incubations was 1%. In addition, emodin, atazanavir, and diclofenac were considered as positive controls for UGT1A10, UGT1A1, and UGT2B7, respectively. The half-maximal inhibitory concentration (IC_50_) values were determined by nonlinear regression analysis in the presence of serial concentrations of natural compounds in XLGB. Traditionally, when the IC_50_ values were less than 10 μM, the inhibitory effects were moderate, and it was worthy of further inhibitory mechanisms (Gao et al., 2020; Xing et al., 2020).

### Inhibitory mechanism of natural components in XLGB against UGT enzymes

The inhibition kinetic parameters (K_i_) and inhibition type were determined using various concentrations of enzyme-selective substrates (4-MU for UGT1A10; E_2_ for UGT1A1; AZT for UGT2B7) in the presence of different concentrations of natural compounds in XLGB. Competitive inhibition, non-competitive inhibition, and mixed-type inhibition models were used to estimate the K_i_ values by nonlinear regression based on Eqs 6–8, respectively. The models with the smallest Akaike information criterion (AIC) and Schwartz information criterion (SC) values were identified as the best models (Gao et al., 2020; Xing et al., 2020).

The parameter *V* is the velocity of the reaction. [*S*] and [*I*] are the concentrations of selective substrates and natural compounds in XLGB, respectively. *K*_i_ represents the affinity between the enzyme and natural compounds. *K*_m_ is the substrate concentration when *V* is 50% of the maximum velocity (*V*_max_). α*K*_i_ is the parameter reflecting the affinity of natural compounds to the complex of enzyme and corresponding specific substrate. When α is far over 1, the binding of herbal compounds could prevent the binding of selective substrate, and the mixed-type inhibition model becomes identical to competitive inhibition.

$$V=\frac{V_{max}\times \left[S\right]}{K_{m}\times \left(1+\frac{\left[I\right]}{K_{i}}\right)+\left[S\right]},$$ (6)

$$V=\frac{V_{max}\times \left[S\right]}{\left(K_{m}+\left[S\right]\right)\times \left(1+\frac{\left[I\right]}{K_{i}}\right)},$$ (7)

$$V=\frac{V_{max}\times \left[S\right]}{K_{m}\times \left(1+\frac{\left[I\right]}{K_{i}}\right)+\left[S\right]\times \left(1+\frac{\left[I\right]}{\alpha K_{i}}\right)}.$$ (8)

### Statistical analysis

All data were presented as mean ± SD (standard deviation). Mean differences between treatment and control groups were analyzed by a two-tailed Student’s t test. The level of significance was set at *p <* 0.05 (∗), *p <* 0.01 (∗∗), or *p <* 0.001 (∗∗∗).

## Results

### Selection of candidate compounds in XLGB

**FIGURE 2:**
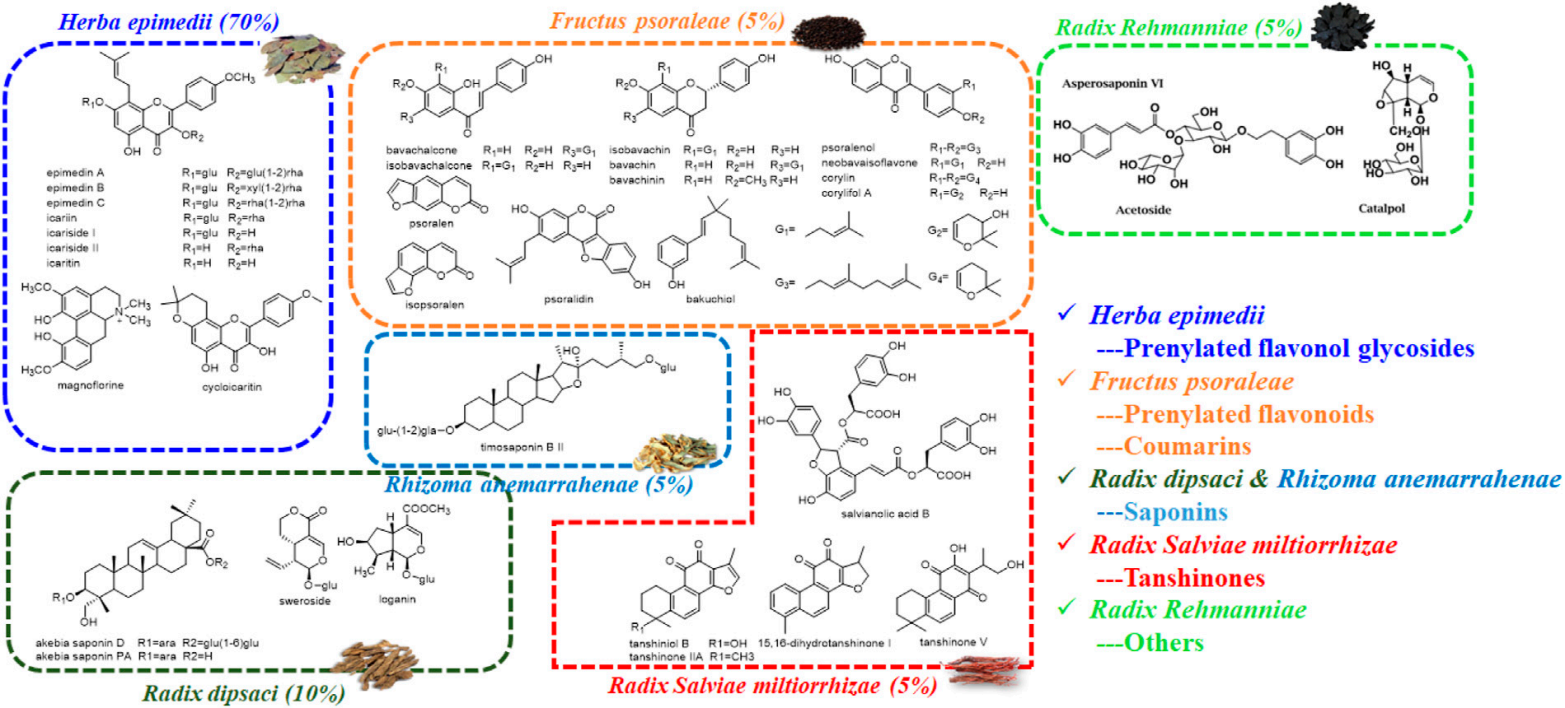
Chemical structures of potential bioactive compounds in XLGB based on its chemical profiles and metabolic profiles.

Four preference principles were as follows: first, they were reported with bone disease-related activity; second, they were the major compounds in chemical profile; third, they were absorbed *in vivo*; fourth, they were the quantitative markers for individual herbs in XLGB according to the Pharmacopoeia of the Peoples’ Republic of China (2020 edition). Therefore, 30 candidate compounds including prenylated flavanol glycosides from *Epimedium brevicornu* Maxim., prenylated flavonoids and coumarins from *Psoralea corylifolia* L., saponins from *Dipsacus asper* Wall. ex Henry and *Anemarrhena asphodeloides* Bge., tanshinones from *Salvia miltiorrhiza* Bge., and other compounds from *Rehmannia glutinosa* Libosch. were selected for further exploration (Figure 2).

### Glucuronidation of estrogen by HLM, HIM, and expressed UGT enzymes

After incubation of estrogens with UDPGA-supplemented HLM or HIM, one metabolite (E1-3*O*-G) of E1 and two glucuronides (E2-3*O*-G and E2-17*O*-G) of E2 were obviously detected (Supplementary Figure S1). Notably, although E3 has three hydroxyl groups, two main metabolites (E3-3*O*-G and E3-16*O*-G) and no E3-17*O*-G were obtained in Supplementary Figure S1, which kept in line with a previous study (Sneitz et al., 2013). The K_m_ values for E1, E2, and E3 ranged from 5.79 to 113.2 μM (Table 1). For E1 and E2, the glucuronidation activity at 3-OH by HIM was obviously higher than that by HLM (Table 1). In addition, E3-16*O*-glucuronidation by HIM was significantly lower than that by HLM (Table 1).

**TABLE 1:** Kinetic parameters of E1, E2, and E3 by HLM, HIM, and expressed UGT enzymes (Mean ± SD). All experiments were performed in triplicate (*n* = 3).

Enzyme	Metabolite	*V*_max_ (pmol/min/mg)	*K*_m_ or S_50_ (μM)	*K*_i_ (μM)	h	*CL*_int_ or *CL*_max_ (μL/min/mg)	Model
E1							
HLM	E1-3-*O*-G	39.86 ± 0.89	6.87 ± 0.59	N.A.	N.A.	5.80 ± 0.09	MM
HIM	E1-3-*O*-G	433.8 ± 7.28	8.82 ± 0.37	N.A.	1.63 ± 0.09	25.25 ± 0.24	Hill
UGT1A3	E1-3-*O*-G	214.8 ± 3.77	9.23 ± 0.39	N.A.	2.09 ± 0.16	11.65 ± 0.95	Hill
E2							
HLM	E2-3-*O*-G	535.4 ± 9.59	14.28 ± 0.61	N.A.	2.14 ± 0.18	18.78 ± 0.56	Hill
	E2-17-*O*-G	92.78 ± 1.48	5.79 ± 0.39	N.A.	N.A.	16.02 ± 0.07	MM
HIM	E2-3-*O*-G	906.5 ± 16.79	15.54 ± 0.72	N.A.	1.66 ± 0.11	29.78 ± 0.98	Hill
	E2-17-*O*-G	23.19 ± 0.75	34.08 ± 3.19	N.A.	N.A.	0.68 ± 0.1	MM
UGT1A1	E2-3-*O*-G	754.9 ± 15.14	11.08 ± 0.48	N.A.	2.43 ± 0.25	34.60 ± 1.42	Hill
UGT1A10	E2-3-*O*-G	927.9 ± 312.4	113.2 ± 46.66	71.98 ± 35.77	N.A.	8.19 ± 4.36	SI
	E2-17-*O*-G	35.71 ± 2.17	16.39 ± 3.53	N.A.	N.A.	2.17 ± 0.22	MM
E3							
HLM	E3-3-*O*-G	136.2 ± 9.5	86.1 ± 12.39	N.A.	N.A.	1.58 ± 0.16	MM
	E3-16-*O*-G	6459 ± 91.92	11.91 ± 0.60	N.A.	N.A.	542.32 ± 0.05	MM
HIM	E3-3-*O*-G	538.7 ± 19.29	50.18 ± 4.40	N.A.	N.A.	10.74 ± 0.10	MM
	E3-16*O*-G	1268 ± 15.06	11.59 ± 0.49	N.A.	N.A.	109.4 ± 0.04	MM
UGT2B7	E3-16-*O*-G	7877 ± 150.7	9.92 ± 0.69	N.A.	N.A.	793.8 ± 0.07	MM

Thirteen expressed UGT enzymes were tested with three estrogens (2 and 10 μM) by a UDPGA-supplemented incubation system. As shown in Figure 2, UGT1A1, 1A3, 1A7, 1A8, 1A9, 1A10, and 2B7 contributed more to the glucuronidation of estrogens. Among them, UGT1A3, 1A1, and 2B7 were the major contributors to the glucuronidation of E1, E2, and E3, respectively (Supplementary Figure S2). However, for E1, the glucuronidation activity of estrone in the intestine was significantly higher than that in the liver (Supplementary Figure S1; Table 1), which was inconsistent with the results that UGT1A3 contributed more to E1-3*O*-glucuronidation due to the predominant expression of UGT1A3 in the liver. The main reason may be the different catalytic activities of commercially recombinant UGT1A10 enzyme and actual intestinal UGT1A10. A previous study has proved that UGT1A10 exhibited efficient glucuronidation to E1 and E2. Therefore, UGT1A10, 1A1, and 2B7 were identified as the most important isozymes for E1, E2, and E3, respectively.

### Optimization of the incubation system

4-MU, β-estradiol, and AZT were selected as specific substrates for UGT1A10, 1A1, and 2B7, respectively. To obtain optimal incubation conditions, the concentration and pH values of Tris-HCl buffer, detergents, MgCl_2_ concentration, β-glucuronidase inhibitors, UDPGA concentration, protein concentration, and incubation time were separately tested (Supplementary Figures S3–S5). On the premise of less than 10% of substrate was metabolized, we got different incubation systems for UGT1A10, 1A1, and 2B7, respectively (Supplementary Table S2). For UGT1A10 (Supplementary Figure S3) and UGT2B7 (Supplementary Figure S5) incubation systems, the glucuronidation activity showed no significant differences in the absence and presence of detergents and β-glucuronidase inhibitors. Therefore, there were no detergents and β-glucuronidase inhibitors in their incubation conditions (Supplementary Table S2).

### Inhibitory effects toward UGT1A10, 1A1, and 2B7 isozymes

**FIGURE 3:**
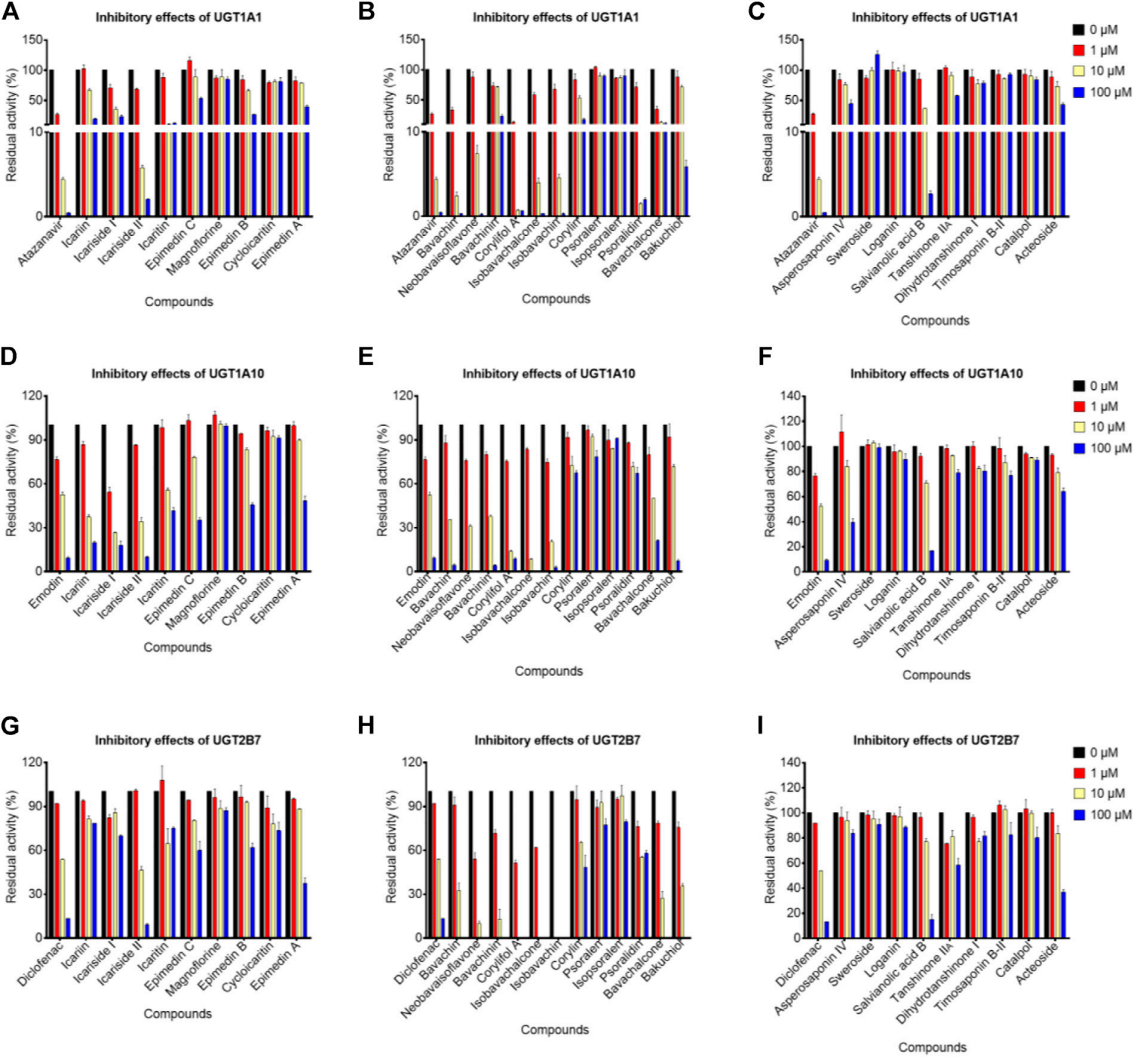
Inhibitory effects of potential bioactive compounds toward UGT1A1, UGT1A10, and UGT2B7 enzymes. **(A)**, **(D)**, and **(G)** represented the inhibitory effects of prenylated flavanol glycosides from *Epimedium brevicornu* Maxim.; **(B)**, **(E)**, and **(H)** represented the inhibitory effects of prenylated flavonoids and coumarins from *Psoralea corylifolia* L.; **(C)**, **(F)**, and **(I)** represented saponins from *Dipsacus asper* Wall. ex Henry and *Anemarrhena asphodeloides* Bge., tanshinones from *Salvia miltiorrhiza* Bge., and other compounds from *Rehmannia glutinosa* Libosch. The probe substrates were incubated at 37°C in the absence (control, 0 μM) and presence of tested compounds (1, 10, and 100 μM). Emodin, atazanavir, and diclofenac were considered as positive controls for UGT1A10, UGT1A1, and UGT2B7, respectively. Data were represented as the mean ± standard deviation of triplicate.

Notably, emodin, atazanavir, and diclofenac (10 μM) exhibited moderate inhibitory effects against UGT1A10, UGT1A1, and UGT2B7 with the remaining activity of 52.43, 4.38, and 53.86%, respectively (Figure 3). In addition, the residual activities of UGT1A10 for icariin, icariside I, icariside II, bavachin, isobavachin, isobavachalcone, neobavaisoflavone, bavachinin, and corylifol A were 37.31, 26.51, 34.22, 35.63, 20.37, 8.61, 31.07, 37.70, and 13.87% of the negative control upon addition of 10 μM, respectively (Figures 3D–F). The inhibitory effects of UGT1A10 by these nine compounds were in a dose-dependent manner (Supplementary Figure S6), and their IC_50_ values were 5.94, 1.39, 4.74, 5.63, 3.62, 2.80, 3.99, 5.42, and 2.91 μM, respectively (Table 2).

**TABLE 2:** Inhibitory parameters of natural compounds derived from XLGB against UGT1A1, UGT1A10, and UGT2B7 (Mean ± SD). All experiments were performed in triplicate.

Compound	UGT1A1			UGT1A10			UGT2B7			
	IC_50_ (μM)	K_i_ (μM)	Inhibitory type	IC_50_ (μM)	K_i_ (μM)	Inhibitory type	IC_50_ (μM)	K_i_ (μM)	α	Inhibitory type
Bavachin	0.61 ± 0.02	0.40 ± 0.05	Non-competitive	5.63 ± 0.46	2.81 ± 0.21	Competitive	4.97 ± 0.55	0.64 ± 0.16	69.70 ± 6.67	Mixed type
Isobavachin	1.73 ± 0.11	3.35 ± 0.53	Competitive	3.62 ± 0.36	1.81 ± 0.28	Non-competitive	0.11 ± 0.01	0.02 ± 0.01	N.A.	Competitive
Bavachalcone	0.53 ± 0.07	1.10 ± 0.08	Non-competitive	—	—	—	4.65 ± 0.52	1.87 ± 0.20	N.A.	Competitive
Isobavachalcone	1.65 ± 0.15	1.20 ± 0.12	Non-competitive	2.80 ± 0.30	1.66 ± 0.19	Non-competitive	1.38 ± 0.22	0.30 ± 0.07	N.A.	Competitive
Neobavaisoflavone	2.86 ± 0.14	0.78 ± 0.16	Non-competitive	3.99 ± 0.67	2.60 ± 0.23	Competitive	1.17 ± 0.10	0.39 ± 0.03	5.15 ± 2.08	Mixed type
Corylifol A	0.44 ± 0.05	0.44 ± 0.07	Competitive	2.91 ± 0.34	0.78 ± 0.06	Non-competitive	0.88 ± 0.08	0.36 ± 0.07	5.17 ± 3.55	Mixed type
Psoralidin	1.95 ± 0.15	0.98 ± 0.23	Non-competitive	—	—	—	—	—	—	—
Salvianolic acid B	3.78 ± 0.53	2.16 ± 0.21	Competitive	—	—	—	—	—	—	—
Icaritin	1.28 ± 0.26	0.42 ± 0.17	Non-competitive	—	—	—	—	—	—	—
Icariside I	3.28 ± 1.20	6.01 ± 0.47	Non-competitive	1.39 ± 0.33	1.92 ± 0.26	Competitive	—	—	—	—
Icariside II	2.47 ± 0.29	1.18 ± 0.21	Competitive	4.74 ± 0.57	3.26 ± 0.42	Competitive	10.19 ± 1.62	2.02 ± 0.26	N.A.	Competitive
Bavachinin	—	—	—	5.42 ± 0.64	3.29 ± 0.23	Competitive	2.27 ± 0.26	1.11 ± 0.17	N.A.	Competitive
Icariin	—	—	—	5.94 ± 1.24	9.77 ± 0.82	Non-competitive	—	—	—	—
Bakuchiol	—	—	—	—	—	—	4.55 ± 0.43	2.02 ± 0.26	N.A.	Competitive

When the tested compounds were 10 μM, the remaining activities of UGT1A1 for icariside I, icariside II, icaritin (Figure 3A), bavachin, isobavachin, bavachalcone, isobavachalcone, neobavaisoflavone, corylifol A, psoralidin (Figure 3B), and salvianolic acid B (Figure 3C) were 35.46, 5.80, 10.15, 2.43, 4.58, 13.00, 3.97, 7.42, 0.72, 1.48, and 36.19% of the negative control, respectively. Furthermore, the inhibition data were fitted to log (tested compounds) and normalized response equations to obtain the IC_50_ values (Supplementary Figure S7). The IC_50_ values of these eleven tested compounds for UGT1A1 were from 0.44 to 3.78 μM (Table 2).

As shown in Figures 3G–I, when treated with respective icariside II, bavachin, isobavachin, neobavaisoflavone, corylifol A, bavachinin, isobavachalcone, bavachalcone, and bakuchiol (10 μM), the residual activities of UGT1A1 were 46.34, 32.54, 0, 10.16, 0, 13.23, 0, 27.24, and 35.47% of negative control, respectively. Furthermore, concentration-dependent inhibitory curves of these nine compounds toward UGT2B7 were depicted, respectively (Supplementary Figure S8). Their IC_50_ values were 10.19, 4.97, 0.11, 1.17, 0.88, 2.27, 1.38, 4.65, and 4.55 μM, respectively (Table 2). The inhibitory mechanism and corresponding inhibitory parameters of tested compounds were further evaluated for UGT1A10, 1A1, and 2B7 when IC_50_ values were less than 10 μM.

### Inhibition mechanism against UGT1A10, 1A1, and 2B7 isozymes

**FIGURE 4:**
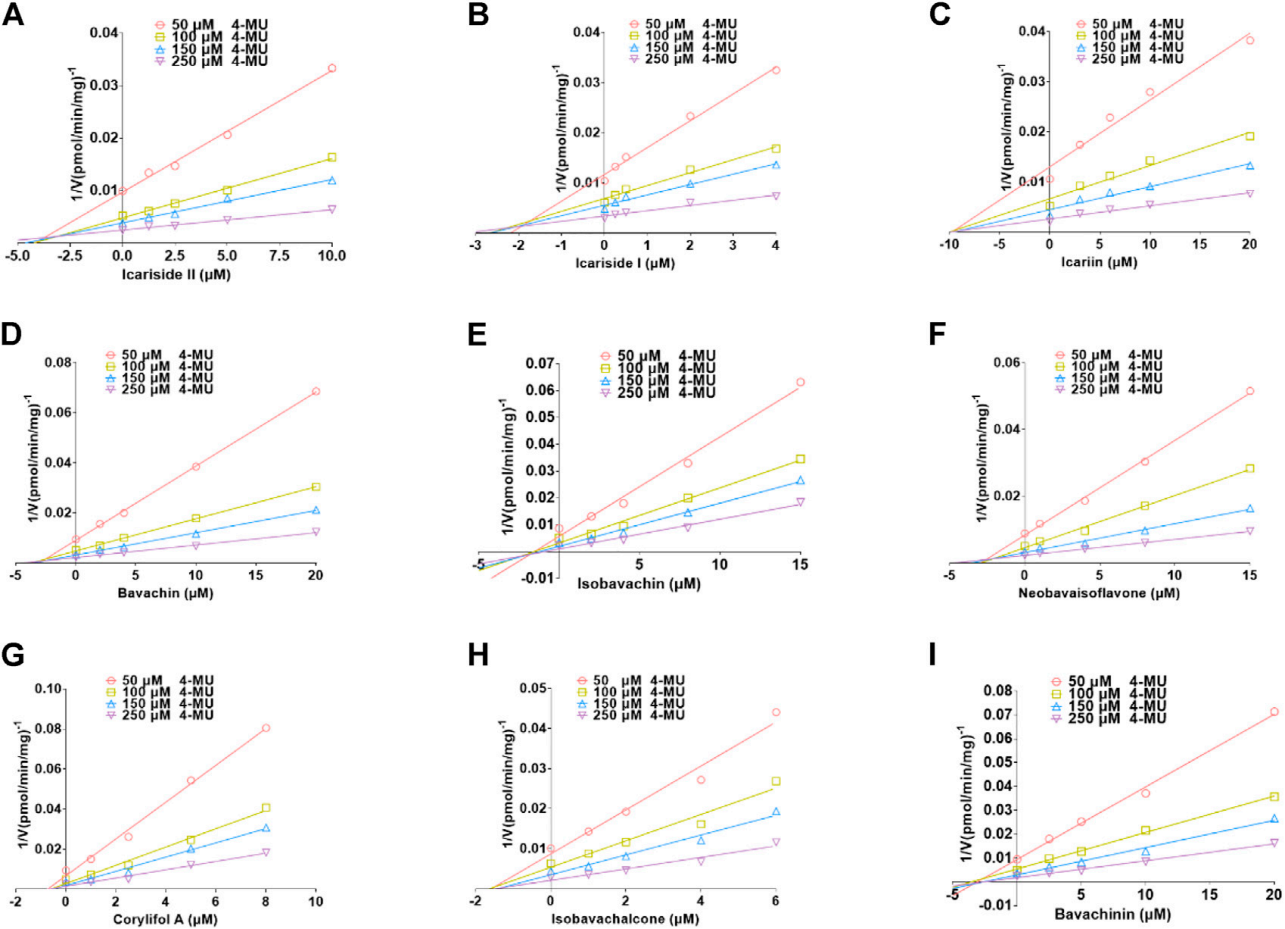
Dixon plots for inhibitory effects of icariside II **(A)**, icariside I **(B)**, icariin **(C)**, bavachin **(D)**, isobavachin **(E)**, neobavaisoflavone **(F)**, corylifol A **(G)**, isobavachalcone **(H)**, and bavachinin **(I)** toward 4-methylumbelliferone-*O*-glucuronidation for UGT1A10. All data were performed in triplicate (mean ± SD).

The plots including the dose-dependent inhibition plot, Lineweaver–Burk plot, Dixon plot, and the secondary plot for determination of K_i_ value for tested compounds toward UGT1A10, 1A1, and 2B7 are shown in Supplementary Figures S9–S11, respectively. In addition, the goodness of fit was determined by the Akaike information criterion (AIC) and *R*2. The AIC and SC values for the UGT1A10 system were obtained after the inhibition data were modeled by three conventional inhibition equations (Supplementary Table S3). Non-competitive inhibition kinetics were observed for icariin, isobavachin, isobavachalcone, and corylifol A against UGT1A10. These findings are kept in line with the Dixon plots for icariside II, icariside I, icariin, bavachin, isobavachin, neobavaisoflavone, corylifol A, isobavachalcone, and bavachinin toward UGT1A10 (Figure 4). The respective K_i_ values were 3.26, 1.92, 9.77, 2.81, 1.81, 2.60, 0.78, 1.66, and 3.29 μM (Table 2).

**FIGURE 5:**
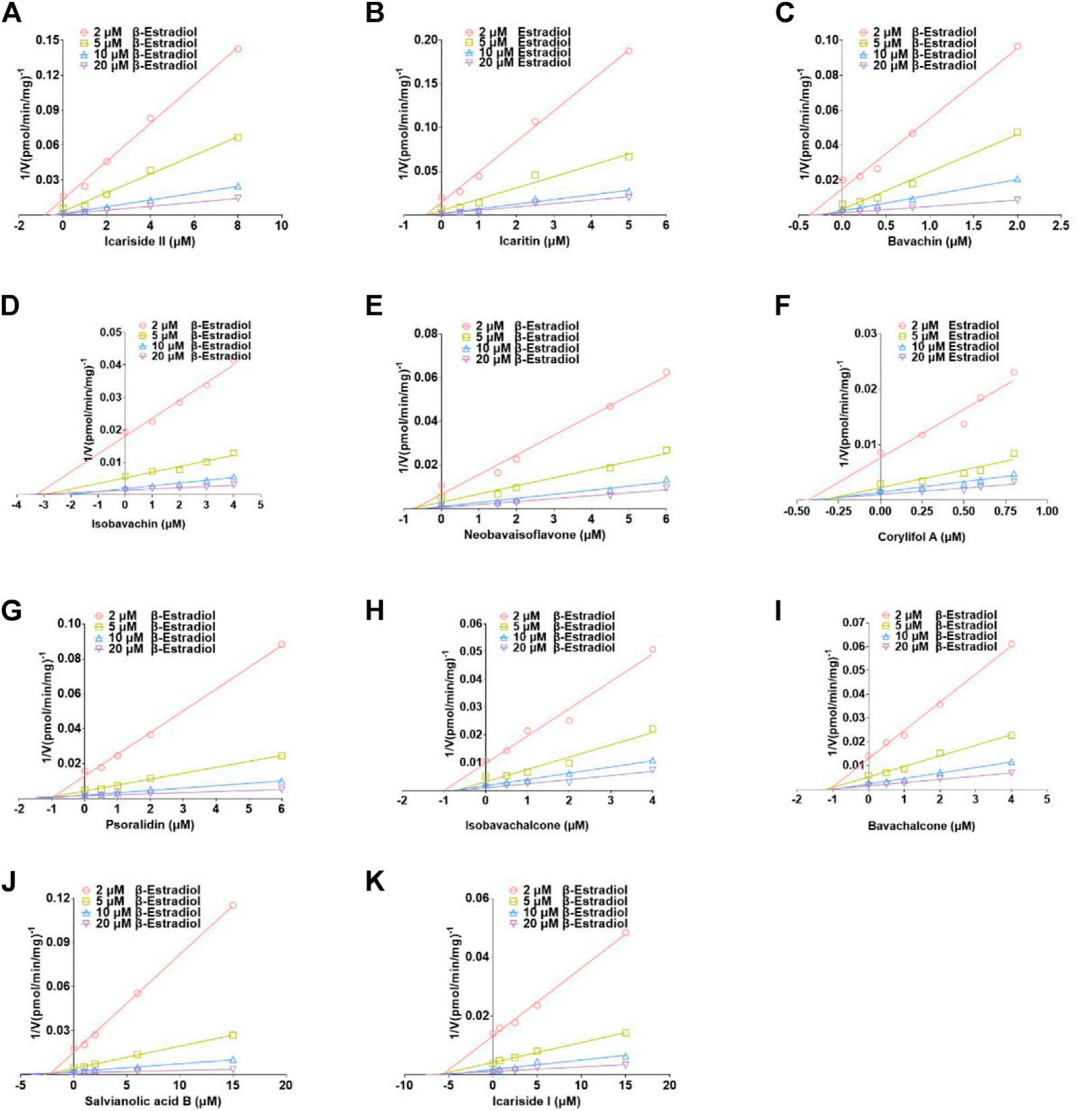
Dixon plots for the inhibitory effects of icariside II **(A)**, icaritin **(B)**, bavachin **(C)**, isobavachin **(D)**, neobavaisoflavone **(E)**, corylifol A **(F)**, psoralidin **(G)**, isobavachalcone **(H)**, bavachalcone **(I)**, salvianolic acid B **(J)**, and icariside I **(K)** against β-estradiol-3-*O*-glucuronidation for UGT1A1. All data represent the mean ± SD of triplicate determinations.

Based on the smallest AIC and SC values (Supplementary Table S4), icariside Ⅱ, isobavachin, corylifol A, and salvianolic acid B demonstrated competitive inhibition mode against UGT1A1, while other compounds exhibited non-competitive inhibition equation toward UGT1A1. Furthermore, the Dixon plots for icariside II (Figure 5A), icaritin (Figure 5B), bavachin (Figure 5C), isobavachin (Figure 5D), neobavaisoflavone (Figure 5E), corylifol A (Figure 5F), psoralidin (Figure 5G), isobavachalcone (Figure 5H), bavachalcone (Figure 5I), salvianolic acid B (Figure 5J), and icariside I (Figure 5K) against UGT1A1 also provided strong evidence to support this judgment. Also, their corresponding K_i_ values were 1.18, 0.42, 0.40, 3.35, 0.78, 0.44, 0.98, 1.20, 1.10, 2.16, and 6.01 μM, respectively (Table 2).

**FIGURE 6:**
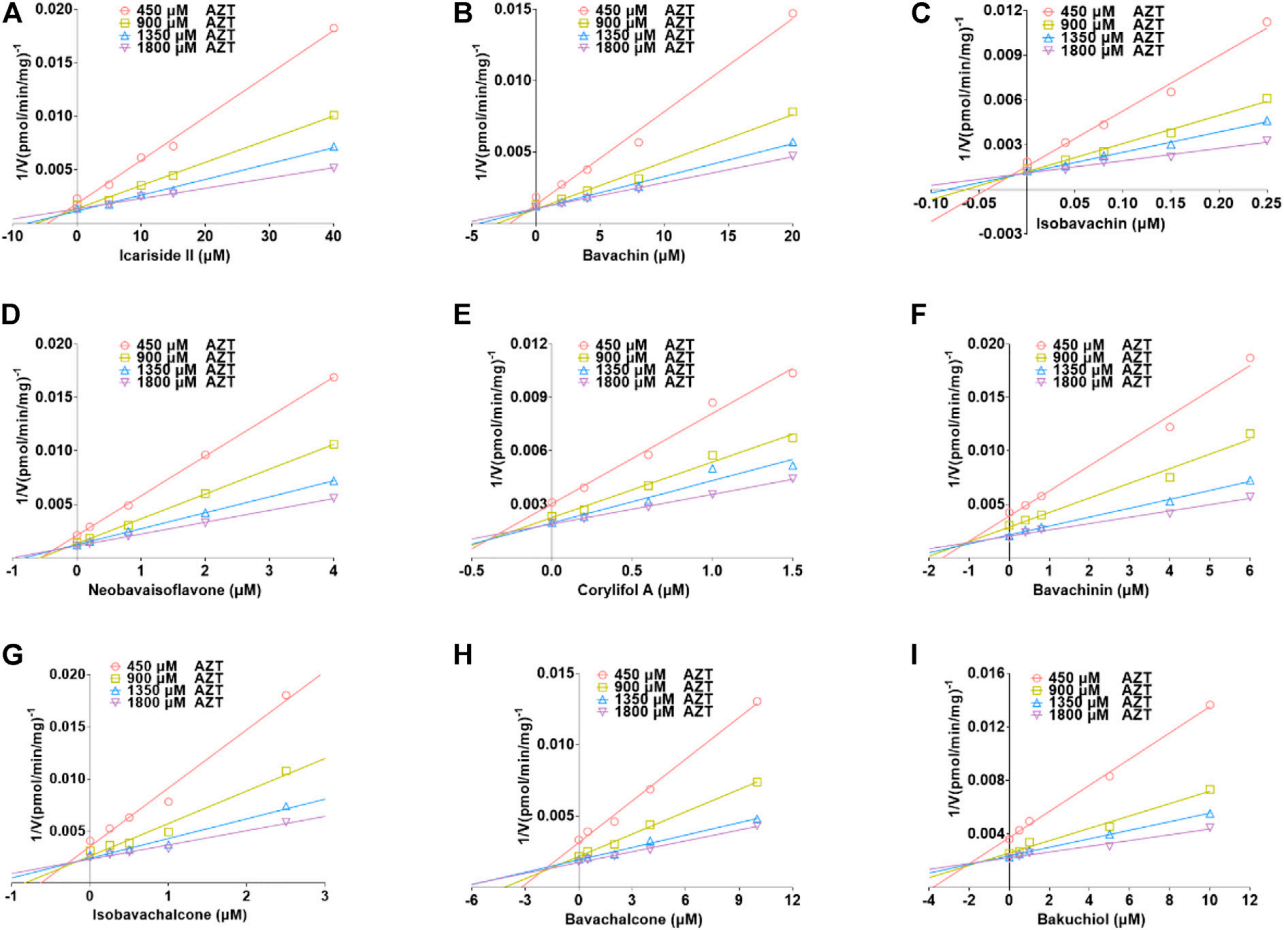
Dixon plots for the inhibitory effects of icariside II **(A)**, bavachin **(B)**, isobavachin **(C)**, neobavaisoflavone **(D)**, corylifol A **(E)**, bavachinin **(F)**, isobavachalcone **(G)**, bavachalcone **(H)**, and bakuchiol **(I)** against zidovudine-*N*-glucuronidation for UGT2B7. All data were shown as means ± SD of triplicate determinations (*n* = 3).

The best models for UGT2B7 are described in Table 2 according to the smallest AIC and SC values (Supplementary Table S5). Icariside Ⅱ (Figure 6A), isobavachin (Figure 6C), bavachinin (Figure 6F), isobavachalcone (Figure 6G), bavachalcone (Figure 6H), and bakuchiol (Figure 6I) exhibited competitive inhibition against UGT2B7, while bavachin (Figure 6B), neobavaisoflavone (Figure 6D), and corylifol A (Figure 6E) displayed non-competitive inhibitory effects toward UGT2B7. Their K_i_ values ranged from 0.02 to 2.02 μM (Table 2). Taken together, these data indicated that prenylated flavanol glycosides and prenylated flavonoids were potent non-selective inhibitors for UGT1A10, 1A1, and 2B7.

## Discussion

XLGB has been successfully used in clinical trials for the prevention and treatment of osteoporosis for over twenty years. It can significantly increase the bone mineral density of lumbar vertebrae in postmenopausal women (Zhu et al., 2012; Li et al., 2018; Chen et al., 2019). The beneficial effects are attributed to the pharmacological properties of phytoestrogens in XLGB (Qin et al., 2005; Mok et al., 2010; Xin et al., 2010). Considering that glucuronidation is one of the most important inactivation pathways of estrogens, and XLGB could markedly elevate the levels of estrogens in OVX rats, we focused on the screening of natural inhibitors that could strongly inhibit the UGT-mediated glucuronidation of estrogens. This is a novel perspective for the discovery of effective compounds in XLGB. In the present study, herbal compounds derived from *Epimedium brevicornu* Maxim. and *Psoralea corylifolia* L. were mainly responsible for the significant inhibitory effects on estrogen glucuronidation.

**FIGURE 7:**
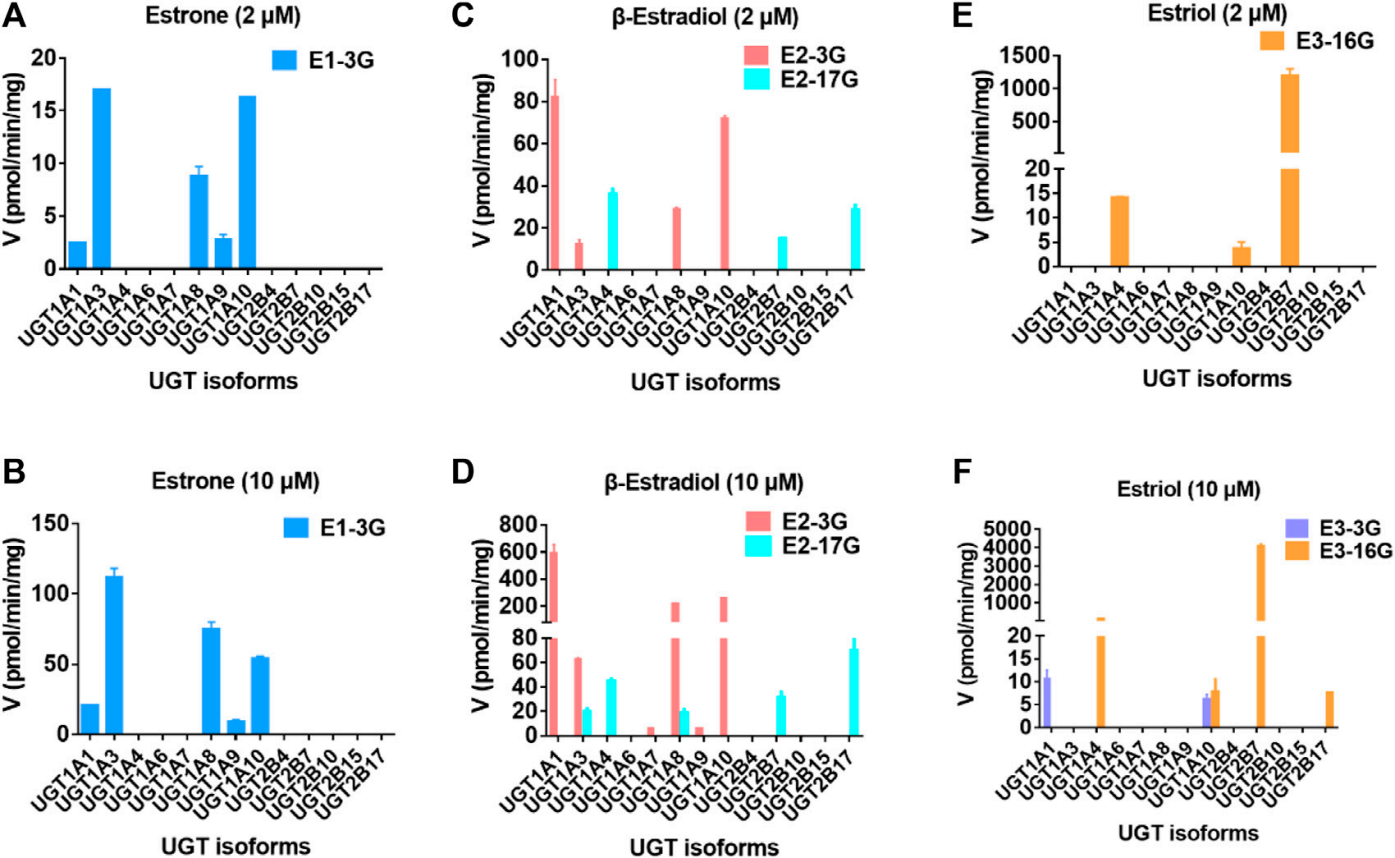
Comparisons of glucuronidation rates of estrone at 2 μM **(A)** and 20 μM **(B)**, β-estradiol at 2 μM **(C)** and 20 μM **(D)**, and estriol at 2 μM **(E)** and 20 μM **(F)** by thirteen expressed UGT enzymes. All experiments were performed in triplicate.

**FIGURE 8:**
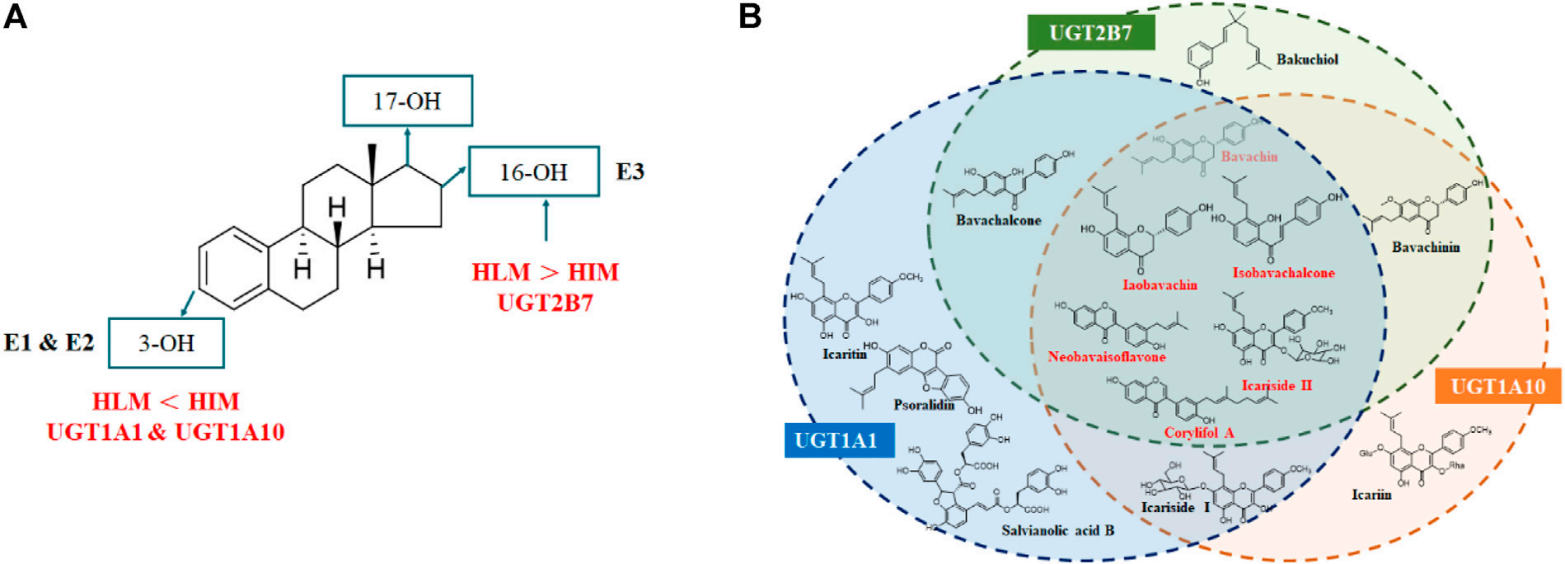
Metabolic characteristics of estrogens by HLM, HIM, and major UGT enzymes **(A)** and identification of natural inhibitors in XLGB against the glucuronidation of estrogens **(B)**.

For the glucuronidation of E3, only two glucuronides (E_3_-3*O*-G and E_3_-16*O*-G) were detected in HLM or HIM incubation systems (Figure 7), which was consistent with the previous study (Sneitz et al., 2013). In addition, several research studies have shown that E_1_-3*O*-G, E_2_-3*O*-G, and E_3_-16*O*-G were the main glucuronide conjugates of E_1_, E_2,_ and E_3_ in urine, respectively (Tikkanen 1973; Van der Berg et al., 2020). Also, only E_1_-3*O*-G and E_2_-3*O*-G could be detected in postmenopausal women's serum (Caron et al., 2009), which kept in line with our resulting *in vitro* glucuronidation assays (Figure 7). Furthermore, microsomes and expressed UGT enzymes demonstrated different regioselectivity of estrogen glucuronidation. For example, HIM exhibited higher catalytic activity for glucuronidation at 3-OH of estrogens than HLM (Table 1; Figure 8A). In contrast, HLM showed more efficient binding to 16-OH and 17-OH of estrogens than HIM. Similarly, UGT1A1 and UGT1A10 displayed higher catalytic activity toward the 3-OH group of estrogens, while UGT2B7 mainly mediated the 17-OH glucuronidation of estrogens (Table 1; Figure 8A). This may be attributed to the differences in the species and abundances of UGT isozymes in the liver and intestines (Yang et al., 2017).

β-estradiol and zidovudine were the widely recognized substrates for UGT1A1 and 2B7, respectively (Xing et al., 2020). So far, no well-accepted probe substrates of UGT1A10 were reported. In this study, 4-MU was used as the substrate for UGT1A10 as described previously (Gao et al., 2020). Different substrates of UGT1A1 could get different inhibitory mechanisms and IC_50_ values (Wang et al., 2015a; Xing et al., 2020). Notably, the inhibition models and obtained IC_50_ values of bavachin, neobavaisoflavone, and isobavachalcone in this study were markedly different from those in the previous study (Wang et al., 2015a). The reason was that β-estradiol, 4-MU, and N-(3-carboxy propyl)-4-hydroxy-1,8-naphthalimide (NCHN) were all used as substrates of UGT1A1 in independent assays. In depth, this was attributed to the existence of three or more binding sites of UGT1A1, β-estradiol, NCHN, and 4-MU may bind with the different regions or subdomains of UGT1A1 (Wang et al., 2015a).

In addition, the structure–activity relationship of tested compounds in XLGB toward the inhibitory effects of estrogen glucuronidation was discussed. It can be obviously found that most prenylated flavonoids and flavonol glycosides have strong inhibitory activities against UGT1A1, 1A10, and 2B7. For instance, icaritin (aglycone of icariside I and icariside II) has higher inhibitory activity on UGT1A1 than icariside I and icariside II, indicating that the number and type of sugar substituent could significantly affect the inhibitory effects (Table 2). For dihydroflavones, the compounds with isopentenyl unit at the C-6 position (bavachin) exhibited stronger inhibition toward UGT1A1 than those with the isopentenyl group at the C-8 position (isobavachin) (Table 2). Compared with bavachin, methylation of C7-OH (bavachinin) would markedly decrease the inhibitory effects, which is kept in line with the previous study (Wang et al., 2015a). Although this study used different substrates (NCHN and 4-MU) of UGT1A1 (Wang et al., 2015a), these compounds exhibited similar inhibitory patterns toward UGT1A1. For chalcones, the position of the isopentenyl unit (bavachalcone and isobavachalcone) had almost no effect on inhibitory activity against UGT1A1. For isoflavones, two isopentenyl units at C-5′ position (corylifol A) increased the inhibitory effects than the compounds with one isopentenyl group at C-5′ position (neobavaisoflavone). This characteristic was also the same as their inhibitory effects toward UGT1A1-mediated 4-MU-glucuronidation and NCHN-glucuronidation (Wang et al., 2015a). Similarly, the numbers and positions of the isopentenyl unit, methyl unit, and sugar group also influenced the inhibitory effects against UGT1A10 and UGT2B7. Taken together, bavachin, neobavaisoflavone, and corylifol A all showed potent inhibitory effects toward UGT1A1, 1A10, and 2B7.

Further, a previous study has demonstrated that it failed to induce observable liver injury after intragastric administration of XLGB at dosages of 1,000 mg/kg (equivalent to 3.3 times the human dose) for 26 weeks in normal rats (Cheng et al., 2013) or up to 1,800 mg/kg/day (equivalent to six times the daily recommended dose) for 26 weeks in OVX rats (Wu et al., 2017). However, many cases of liver injury associated with XLGB have emerged in China recently. Therefore, CFDA has also warned of the risks of liver damage from XLGB in 2016 and turned this drug from over-the-counter treatment to prescription. Furthermore, when lipopolysaccharide (LPS) and XLGB were co-administrated to OVX rats, marked hepatotoxicity was observed (Wu et al., 2019; Li et al., 2020). Also, mild immune stress, disrupted lipid metabolism, extensive liver necrosis and inflammatory infiltration, apoptosis, and expression of oxidative stress-related proteins may be associated with XLGB-induced hepatotoxicity in humans (Wu et al., 2019; Li et al., 2020). Meanwhile, eight endogenous components including sphinganine, glycerophosphoethanolamine, and phenylalanine were identified as the potential biomarkers for XLGB-induced liver injury (Li et al., 2020).

*Psoralea corylifolia* L., an important herb of XLGB, has been widely used for the treatment of bone fracture and osteoporosis (Li et al., 2017). However, several clinical cases of acute liver injury after oral administration of *Psoralea corylifolia* L. have been reported (Teschke and Bahre, 2009; Wang et al., 2020). This hepatotoxicity may involve in the destruction of the biosynthesis and transportation of bile acid, whereas bakuchiol was identified as the important component for cholestatic hepatotoxicity (Li et al., 2017). Therefore, herbal compounds in *Psoralea corylifolia* L. contribute more to the hepatotoxicity of XLGB.

On the other hand, drug–drug interactions (DDI) usually cause significant clinical safety issues due to the inhibition or activation of drugs (or herbal compounds) toward clinically important CYP or UGT enzymes (Gao et al., 2020; Xing et al., 2020). Clinical acute liver injury induced by XLGB was usually accompanied by markedly elevated bilirubin (Wu et al., 2019). Bilirubin is an important index for the clinical judgment of jaundice and liver function. UGT1A1 is the sole enzyme responsible for the clearance of bilirubin. This is the reason why bilirubin-*O*-glucuronidation is always used as the specific probe reaction for the evaluation of the potential for drug/herb-drug interactions mediated by UGT1A1 in human tissues (Wang et al., 2015a). In this respect, bavachin and corylifol A showed strong inhibitory effects against UGT1A1 (K_i_ < 1 μM), while neobavaisoflavone, isobavachalcone, and bavachinin were identified as moderate inhibitors with K_i_ values ranging from 1.61 to 9.86 μM (Wang et al., 2015a). Several other herbal compounds in *Psoralea corylifolia* L. (isobavachin, norbakuchinic acid, psoralidin, bavachinin, etc.) also exhibited different degrees of inhibitory activity toward UGT1A1 (Sun et al., 2015; Xing et al., 2020). These findings indicated *Psoralea corylifolia* L.-containing herbal compounds may be the important reason for triggering hepatoxicity, including elevated bilirubin levels and liver injury (Wang et al., 2015a).

Except for endogenous substances, UGT1A1 also mediated the glucuronidation of many therapeutic drugs (i.e., SN-38, raloxifene, and irinotecan) (Lv et al., 2019). UGT2B7 appeared to play a particularly important role in the metabolic clearance of several clinical drugs (morphine, zidovudine, lorazepam, etc.) (Kasteel et al., 2020). Also, several compounds including 4-MU could underdo efficient glucuronidation by UGT1A10 (Gao et al., 2020). In this study, our results showed that prenylated flavonoid analogs were strong inhibitors against UGT1A1, 1A10, and 2B7 with K_i_ values less than 1.0 μM. These findings suggested that more attention should be paid to avoiding clinical adverse issues (acute liver injury, drug toxic effect, etc.) due to DDI after co-administration.

Moreover, there were several limitations in this study. For example, except glucuronidation, SULT1E1-mediated sulfation is another important inactivation pathway of estrogen (Van der Berg et al., 2020). Hence, the interaction (inhibition or induction) between herbal compounds in XLGB and SULT enzymes needed to be explored. In addition, the glucuronides of prenylated compounds were excreted by efflux transporter including breast cancer resistance protein (BCRP) and multidrug resistance-associated proteins (MRPs) (Li et al., 2020a; Li et al., 2020b; Qin et al., 2021). Likewise, BCRP and MRPs are also the main contributors to the excretion of estrogen glucuronide (Jarvinen et al., 2018). Whether this will affect the efflux and level of estrogens needs to be further studied. Moreover, the quantitative analysis of estrogens and their related glucuronides have been developed and validated in our laboratory (Supplementary Table S1; Tang et al., 2020). However, only the *in vivo* levels of E_1_ and E_2_ were measured after oral administration of XLGB in OVX-mice serum, because E3 is too low to be detected (Tang et al., 2020). Meanwhile, the *in vivo* levels of estrogen-related glucuronides in OVX-mice serum and liver were absent, and this also urges us to further investigate the levels of glucuronides and mRNA and protein levels of related UGT enzymes in OVX-mice tissues. It is also necessary to explore the changes in estrogens and their glucuronides after intragastric administration of bavachin, neobavaisoflavone, and corylifol A, respectively. This would benefit the understanding of effective components in XLGB for the increase of estrogens through regulating the glucuronidation of estrogens.

## Conclusion

In conclusion, UGT1A10, 1A1, and 2B7 contributed more to the glucuronidation of estrogen. Based on the optimal incubation system, fourteen compounds demonstrated potent inhibitory effects against UGT1A10, 1A1, and 2B7. Among them, icariside II, bavachin, isobavachin, isobavachalcone, neobavaisoflavone, and corylifol A simultaneously exhibited strong inhibitory effects toward UGT1A10, 1A1, and 2B7 with IC_50_ values ranging from 0.11 to 10.19 μM (Figure 8B). Taken together, prenylated flavanols from *Epimedium brevicornu* Maxim. and prenylated flavonoids from *Psoralea corylifolia* L. contributed more to inhibiting the glucuronidation of estrogen. This study provides some new insights into the identification of effective compounds of XLGB.

## Data Availability

The datasets presented in this study can be found in online repositories. The names of the repository/repositories and accession number(s) can be found in the article/Supplementary Material.

## Abbreviations

4-MU, 4-methylumbelliferone; AZT, zidovudine; E1, estrone; E2, β-estradiol; E3, estriol; HIM, human intestine microsomes; HLM, human liver microsomes; MRM, multiple reaction monitoring; NCHN, N-(3-carboxy propyl)-4-hydroxy-1,8-naphthalimide; OVX, ovariectomized; UDPGA, uridine diphosphate glucuronic acid; UGT, UDP-glucuronosyltransferases; UHPLC, ultra-high performance liquid chromatography; XLGB, Xian-Ling-Gu-Bao.
