# Supplementary material for: Identification of naturally occurring inhibitors in Xian-Ling-Gu-Bao capsule against the glucuronidation of estrogens
Source: Front Pharmacol. 2022 Aug 4;13:935685. doi: 10.3389/fphar.2022.935685 (PMC9386001; doi:10.3389/fphar.2022.935685)
Supplement: Supplementary file 1 [file DataSheet1.docx]

**Identification of naturally occurring inhibitors in Xian-Ling-Gu-Bao capsule against the glucuronidation of estrogens**

Liangliang He ^1, †^, Chunxia Xu ^1, †^, Ziying Wang ^2^, Shuyi Duan ^3^, Jinjin Xu ^1^, Chuan Li ^4^, Xinsheng Yao ^1, 5^, Frank J. Gonzalez ^6^, Zifei Qin ^1, 3,^ *, Zhihong Yao ^1, 4, 5,^ *

^1^ College of Pharmacy, Jinan University, Guangzhou 510632, China;

^2^ School of Chemistry, University of Bristol, Cantock's Close, Bristol BS8 1TS, UK;

^3^ Department of Pharmacy, the First Affiliated Hospital of Zhengzhou University, Zhengzhou 450052, China;

^4^ State key Laboratory of Drug Research, Shanghai Institute of Materia Medica, Chinese Academy of Sciences, Shanghai 201203, China;

^5^ International Cooperative Laboratory of Traditional Chinese Medicine Modernization and Innovative Drug Development Ministry of P.R. China, Jinan University, Guangzhou 510632, China;

^6^ Laboratory of Metabolism, Centre for Cancer Research, National Cancer Institute, National Institutes of Health, Bethesda, MD, USA.

^†^ These authors contributed equally to this work.

***Correspondence:**

*E-mails*: yaozhihong_jnu@163.com (Zhihong Yao);

qzf1989@163.com (Zifei Qin);

**Table lists and Figure caption**

**Table S1** Multiple reaction monitoring parameters for E1, E2, E3 and their glucuronides.

**Table S2** Optimal incubation system for UGT1A1, 1A10 and 2B7.

**Table S3** Selection of inhibition models of human recombinant UGT1A10 by nine active components from XLGB.

**Table S4** Selection of inhibition models of human expressed UGT1A1 by eleven active components from XLGB.

**Table S5** Selection of inhibition models of human expressed UGT2B7 by nine active components from XLGB.

**Figure S1** Kinetic profiles for estrone-*O*-glucuronidation (A) by HLM, β-estradiol-*O*-glucuronidation (B) by HLM, estriol-*O*-glucuronidation (C) by HLM, and estrone-*O*-glucuronidation (D) by HIM, β-estradiol-*O*-glucuronidation (E) by HIM, estriol-*O*-glucuronidation (F) by HIM. In each panel, the insert figure showed the corresponding Eadie-Hofstee plot. All experiments were performed in triplicate (n = 3).

**Figure S2** Kinetic profiles for estrone-*O*-glucuronidation by UGT1A3 (A), β-estradiol-*O*-glucuronidation (B) by UGT1A1, β-estradiol-*O*-glucuronidation (C) by UGT1A10 and estriol-*O*-glucuronidation (D) by UGT2B7. In each panel, the insert figure showed the corresponding Eadie-Hofstee plot. All experiments were performed in triplicate (n = 3).

**Figure S3** Effects of different incubation conditions for 4-MU-glucuronidation by UGT1A10 (mean ± SD, n = 3). (A) different concentration of Tris-HCl buffer, (B) pH values of Tris-HCl solution, (C) detergents, (D) MgCl_2_ concentration, (E) β-glucuronidase inhibitors, (F) UDPGA concentration, (G) protein concentration, (H) incubation time. All experiments were performed in triplicate (n = 3). (* *p* < 0.05, ** *p* < 0.01, *** *p* < 0.001)

**Figure S4** Incubation condition optimization involving in different concentration (A) and pH values (B) of Tris-HCl solution, detergents (C), MgCl_2_ concentration (D), β-glucuronidase inhibitors (E), UDPGA concentration (F), protein concentration (G), incubation time (H) for β-estradiol-*O*-glucuronidation by UGT1A1. All experiments were performed in triplicate (n = 3). (* *p* < 0.05, ** *p* < 0.01, *** *p* < 0.001)

**Figure S5** Incubation condition optimization involving in different concentration (A) and pH values (B) of Tris-HCl solution, detergents (C), MgCl_2_ concentration (D), β-glucuronidase inhibitors (E), UDPGA concentration (F), protein concentration (G), incubation time (H) for zidovudine-*N*-glucuronidation by UGT2B7. All experiments were performed in triplicate (n = 3). (* *p* < 0.05, ** *p* < 0.01, *** *p* < 0.001)

**Figure S6** The IC_50_ values of icariside II (A), icariside I (B), icariin (C), bavachin (D), isobavachin (E), neobavaisoflavone (F), corylifol A (G), isobavachalcone (H), and bavachinin (I) against UGT1A10. The data were fit to log (concentration) and normalized response equations. Each data point represented the mean value ± the S.D. of triplicate determination.

**Figure S7** The IC_50_ values of icariside II (A), icaritin (B), bavachin (C), isobavachin (D), neobavaisoflavone (E), corylifol A (F), psoralidin (G), isobavachalcone (H), bavachalcone (I), salvianolic acid B (J), and icariside I (K) against UGT1A1. The data were fit to log (concentration) and normalized response equations. Each data point represented the mean value ± the S.D. of triplicate determination.

**Figure S8** The IC_50_ values of icariside II (A), bavachin (B), isobavachin (C), neobavaisoflavone (D), corylifol A (E), bavachinin (F), isobavachalcone (G), bavachalcone (H), bakuchiol (I) against UGT2B7. The data were fit to log (concentration) and normalized response equations. Each data point represented the mean value ± the S.D. of triplicate determination.

**Figure S9** The inhibitory effects of tested compounds in XLGB on the glucuronidation of 4-MU in UGT1A10. (A) Dose-dependent inhibition of tested compounds in XLGB towards UGT1A10; (B) Lineweaver-Burk plot; (C) Dixon plot; (D) The secondary plot for determination of K_i_ value. All experiments were performed in triplicate.

**Figure S10** The inhibitory effects of tested compounds in XLGB on the glucuronidation of E2 in UGT1A1. (A) Dose-dependent inhibition of tested compounds in XLGB towards UGT1A1; (B) Lineweaver-Burk plot; (C) Dixon plot; (D) The secondary plot for determination of K_i_ value. All experiments were performed in triplicate.

**Figure S11** The inhibitory effects of tested compounds in XLGB on the glucuronidation of AZT in UGT2B7. (A) Dose-dependent inhibition of tested compounds in XLGB towards UGT2B7; (B) Lineweaver-Burk plot; (C) Dixon plot; (D) The secondary plot for determination of K_i_ value. All experiments were performed in triplicate.

**Table S1** Multiple reaction monitoring parameters for E1, E2, E3 and their glucuronides.

| **Compound** | ***t*_R_ (min)** | **Precursor ion (*m/z*)** | **Product ion (*m/z*)** | **CV (V)** | **CE (eV)** |
| --- | --- | --- | --- | --- | --- |
| E_1_ | 5.15 | 271.17 | 253.04 | 20 | 10 |
| E_1_-3*O*-G | 3.61 | 271.17 | 253.04 | 20 | 10 |
| E_2_ | 4.31 | 273.18 | 106.91 | 20 | 25 |
| E_2_-3*O*-G | 3.26 | 273.18 | 106.91 | 25 | 10 |
| E_2_-17*O*-G | 3.41 | 273.18 | 106.91 | 25 | 15 |
| E_3_ | 3.50 | 271.17 | 253.04 | 20 | 10 |
| E_3_-3*O*-G | 3.00 | 271.17 | 253.04 | 20 | 10 |
| E_3_-16*O*-G | 3.30 | 271.17 | 253.04 | 20 | 10 |
| IS (4-MU-G) | 1.66 | 353.08 | 177.05 | 25 | 20 |

**Table S2** Optimal incubation system for UGT1A1, 1A10 and 2B7

| **System** | **UGT1A1** | **UGT1A10** | **UGT2B7** |
| --- | --- | --- | --- |
| Tris-HCl buffer (25 °C) | 50 mM | 100 mM | 100 mM |
| Tris-HCl buffer (25 °C) | pH=8.0 | pH=7.4 | pH=7.4 |
| Detergents | alamethicin (20 μg/mL) | / | / |
| MgCl_2_ solution | 4 mM | 1 mM | 4 mM |
| β-glucuronidase inhibitors | 5 mM | / | / |
| UDPGA | 2 mM | 6 mM | 2 mM |
| Protein concentration | 25 µg/mL | 50 µg/mL | 25 µg/mL |
| Time | 120 min | 150 min | 180 min |

**Table S3** Selection of inhibition models of human recombinant UGT1A10 by nine active components from XLGB.

| **Compound** | **Type**  **of inhibition** | ***R^2^*** | **AIC** | **SC** | **Selection**  **of model** |
| --- | --- | --- | --- | --- | --- |
| icariside II | Competitive  Noncompetitive  Uncompetitive  Mixed model | 0.990  0.988  0.957  0.989 | 159.00  161.73  187.16  161.00 | 161.99  164.72  190.15  164.98 | **√** |
| icariside I | Competitive  Noncompetitive  Uncompetitive  Mixed model | 0.968  0.967  0.948  0.968 | 171.87  172.35  181.56  173.87 | 174.85  175.33  184.55  177.85 | **√** |
| icariin | Competitive  Noncompetitive  Uncompetitive  Mixed model | 0.982  0.982  0.939  0.982 | 173.30  173.25  197.86  175.25 | 176.29  176.24  200.84  179.23 | **√** |
| bavachin | Competitive  Noncompetitive  Uncompetitive  Mixed model | 0.996  0.995  0.964  0.996 | 143.20  145.40  186.53  145.58 | 146.19  148.39  189.52  149.57 | **√** |
| isobavachin | Competitive  Noncompetitive  Uncompetitive  Mixed model | 0.980  0.982  0.954  0.981 | 175.64  174.53  192.49  177.32 | 178.62  177.52  195.47  188.30 | **√** |
| neobavaisoflavone | Competitive  Noncompetitive  Uncompetitive  Mixed model | 0.995  0.994  0.965  0.995 | 148.42  150.67  187.31  150.70 | 151.40  153.66  190.30  154.68 | **√** |
| corylifol A | Competitive  Noncompetitive  Uncompetitive  Mixed model | 0.983  0.984  0.952  0.984 | 170.27  169.94  191.04  171.98 | 173.26  172.93  194.02  175.96 | **√** |
| isobavachalcone | Competitive  Noncompetitive  Uncompetitive  Mixed model | 0.984  0.985  0.946  0.985 | 162.15  161.27  186.55  163.52 | 165.13  164.25  189.54  167.50 | **√** |
| bavachinin | Competitive  Noncompetitive  Uncompetitive  Mixed model | 0.994  0.992  0.947  0.994 | 148.29  154.42  192.39  151.36 | 151.28  157.41  195.38  155.34 | **√** |

**Table S4** Selection of inhibition models of human expressed UGT1A1 by eleven active components from XLGB.

| **Compound** | **Type**  **of inhibition** | ***R^2^*** | **AIC** | **SC** | **Selection**  **of model** |
| --- | --- | --- | --- | --- | --- |
| icariside II | Competitive  Noncompetitive  Uncompetitive  Mixed model | 0.955  0.954  0.916  0.955 | 205.97  206.34  219.42  207.87 | 208.96  209.33  222.40  211.85 | **√** |
| icariside I | Competitive  Noncompetitive  Uncompetitive  Mixed model | 0.969  0.970  0.954  0.969 | 221.97  221.94  230.57  223.91 | 224.96  224.92  233.56  227.89 | **√** |
| icaritin | Competitive  Noncompetitive  Uncompetitive  Mixed model | 0.912  0.915  0.889  0.915 | 215.85  214.95  221.24  216.95 | 218.84  217.94  224.23  220.93 | **√** |
| bavachin | Competitive  Noncompetitive  Uncompetitive  Mixed model | 0.986  0.986  0.951  0.986 | 181.19  181.04  208.88  182.82 | 184.18  184.02  211.87  186.81 | **√** |
| isobavachin | Competitive  Noncompetitive  Uncompetitive  Mixed model | 0.972  0.970  0.949  0.971 | 202.06  203.06  214.83  204.06 | 205.04  206.05  217.81  208.04 | **√** |
| neobavaisoflavone | Competitive  Noncompetitive  Uncompetitive  Mixed model | 0.937  0.943  0.931  0.943 | 216.64  214.47  218.83  214.98 | 219.63  217.45  221.81  218.96 | **√** |
| corylifol A | Competitive  Noncompetitive  Uncompetitive  Mixed model | 0.953  0.952  0.920  0.953 | 216.24  216.88  227.72  217.48 | 219.23  219.86  230.71  221.46 | **√** |
| psoralidin | Competitive  Noncompetitive  Uncompetitive  Mixed model | 0.961  0.962  0.936  0.962 | 209.94  209.80  220.74  211.49 | 212.92  212.79  223.73  215.47 | **√** |
| isobavachalcone | Competitive  Noncompetitive  Uncompetitive  Mixed model | 0.975  0.978  0.959  0.978 | 198.54  195.50  210.48  197.28 | 201.53  198.49  213.47  201.27 | **√** |
| bavachalcone | Competitive  Noncompetitive  Uncompetitive  Mixed model | 0.977  0.979  0.961  0.979 | 190.80  188.51  202.29  190.31 | 193.79  191.50  205.27  194.29 | **√** |
| salvianolic acid B | Competitive  Noncompetitive  Uncompetitive  Mixed model | 0.987  0.985  0.955  0.987 | 190.80  194.65  217.36  193.80 | 194.79  197.64  220.35  197.78 | **√** |

**Table S5** Selection of inhibition models of human expressed UGT2B7 by nine active components from XLGB.

| **Compound** | **Type**  **of inhibition** | ***R^2^*** | **AIC** | **SC** | **Selection**  **of model** |
| --- | --- | --- | --- | --- | --- |
| Icariside II | Competitive  Noncompetitive  Uncompetitive  Mixed model | 0.980  0.977  0.945  0.947 | 199.26  201.98  219.09  220.86 | 202.75  204.97  222.08  224.85 | **√** |
| bavachin | Competitive  Noncompetitive  Uncompetitive  Mixed model | 0.988  0.984  0.956  0.993 | 195.73  201.75  222.75  188.64 | 198.72  204.74  225.15  192.62 | **√** |
| isobavachin | Competitive  Noncompetitive  Uncompetitive  Mixed model | 0.986  0.958  0.910  0.986 | 242.05  218.15  233.45  258.95 | 245.04  221.13  236.44  262.93 | **√** |
| neobavaisoflavone | Competitive  Noncompetitive  Uncompetitive  Mixed model | 0.998  0.993  0.970  0.998 | 159.92  188.64  216.47  155.66 | 162.90  191.63  219.45  159.65 | **√** |
| corylifol A | Competitive  Noncompetitive  Uncompetitive  Mixed model | 0.983  0.974  0.939  0.986 | 177.65  185.86  202.88  175.24 | 180.65  188.85  205.87  179.22 | **√** |
| bavachinin | Competitive  Noncompetitive  Uncompetitive  Mixed model | 0.979  0.977  0.957  0.979 | 183.90  185.59  198.31  185.46 | 186.89  188.57  201.30  189.44 | **√** |
| isobavachalcone | Competitive  Noncompetitive  Uncompetitive  Mixed model | 0.980  0.964  0.931  0.980 | 171.00  182.20  195.35  172.98 | 173.98  185.19  198.34  176.96 | **√** |
| bavachalcone | Competitive  Noncompetitive  Uncompetitive  Mixed model | 0.990  0.977  0.945  0.990 | 171.70  187.44  205.01  173.69 | 174.69  190.42  208.00  177.67 | **√** |
| bakuchiol | Competitive  Noncompetitive  Uncompetitive  Mixed model | 0.981  0.959  0.925  0.981 | 174.88  190.43  202.53  176.88 | 177.87  193.41  205.51  180.87 | **√** |


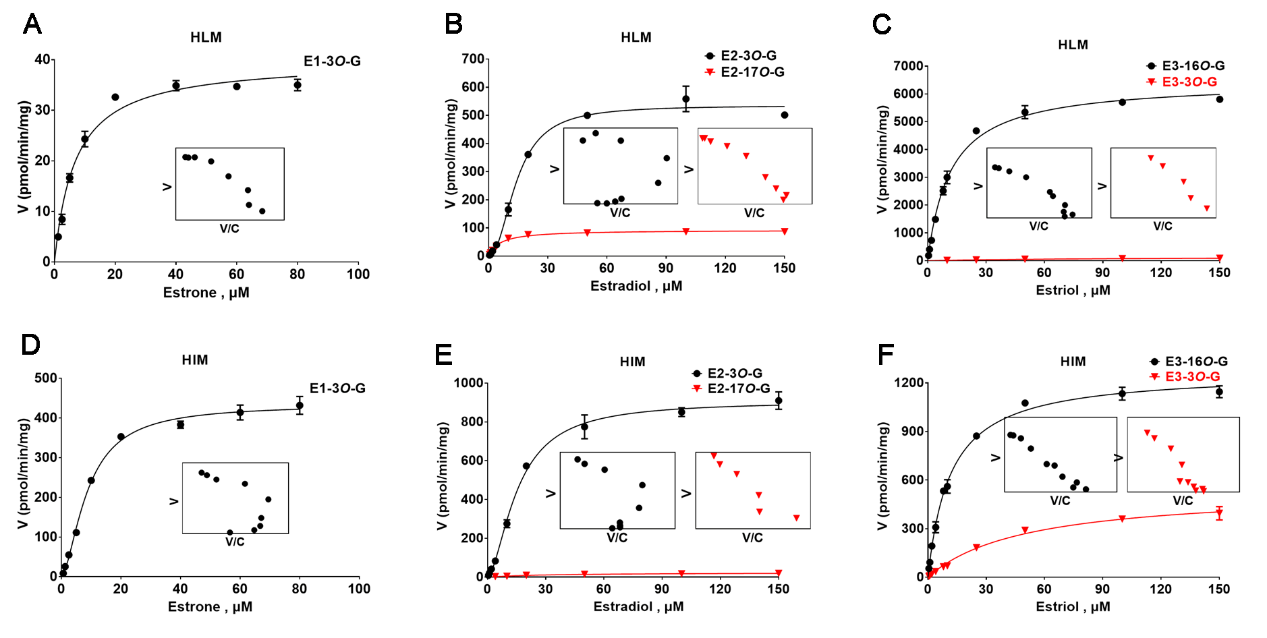


**Figure S1**


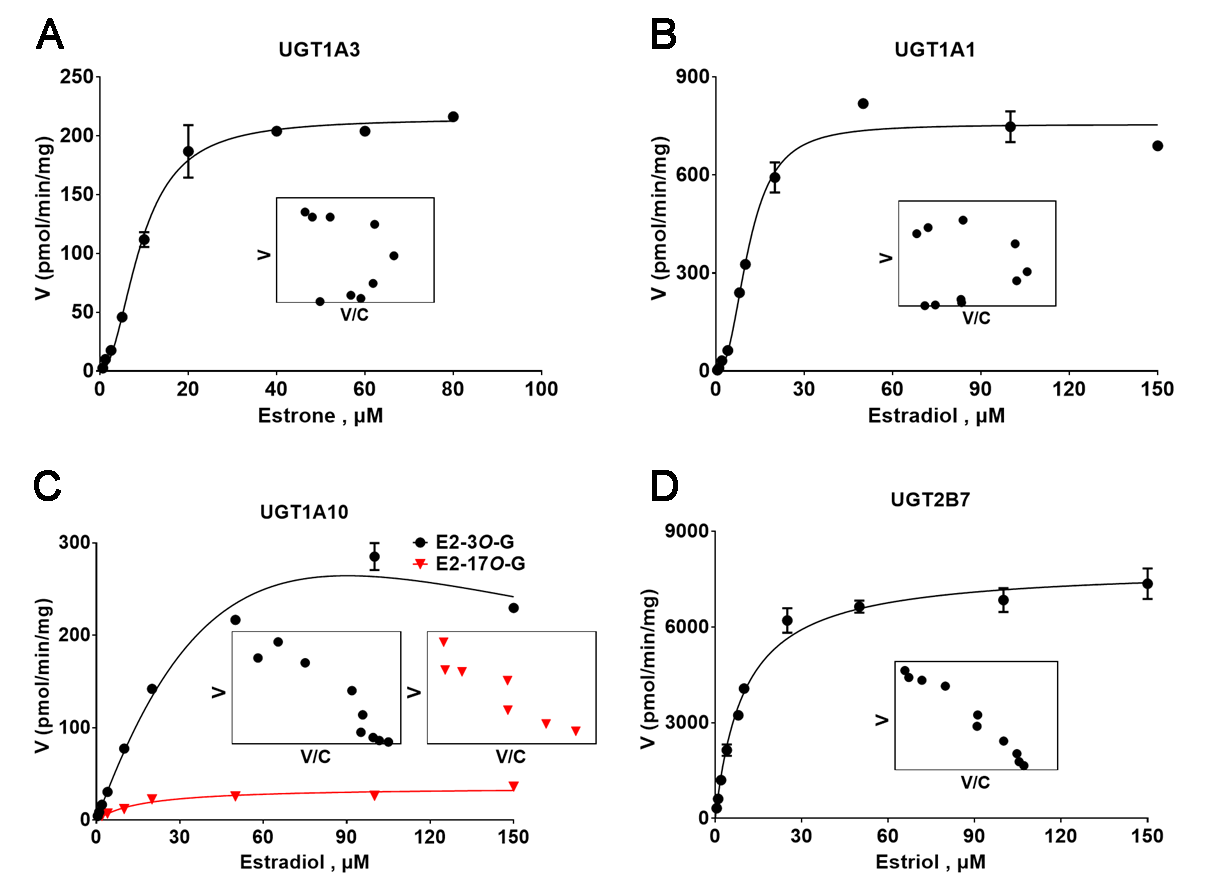


**Figure S2**


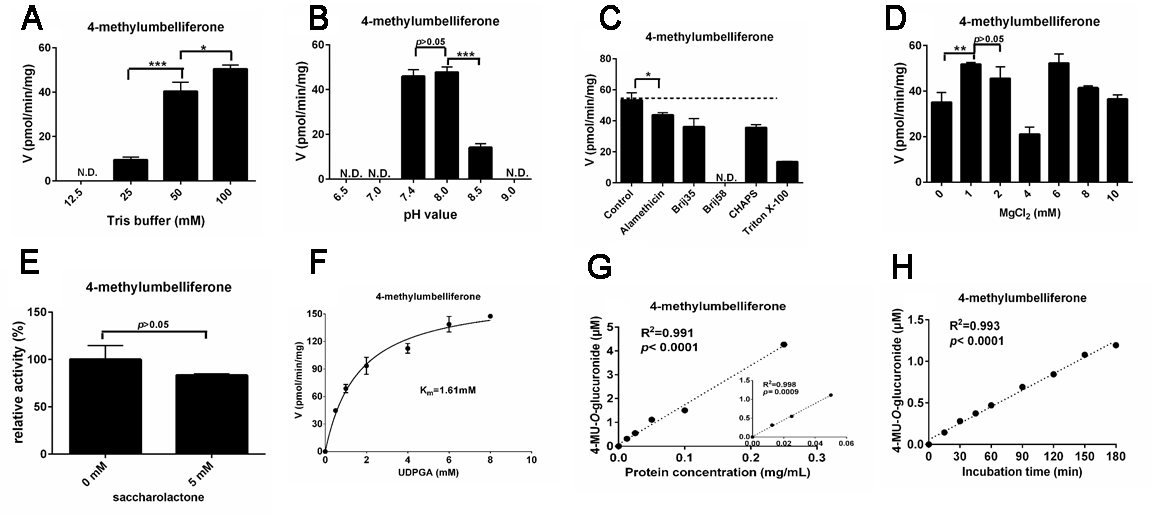


**Figure S3**


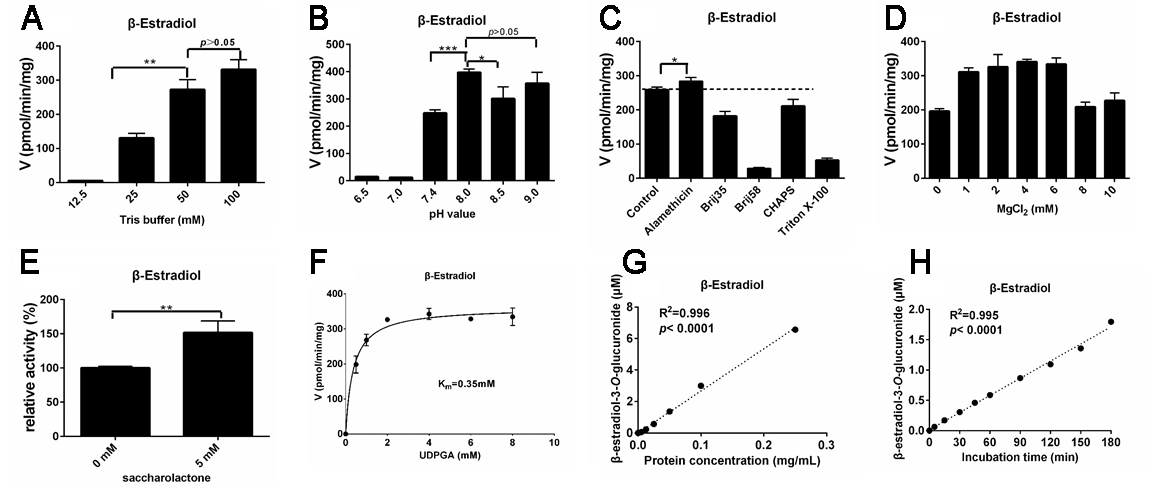


**Figure S4**


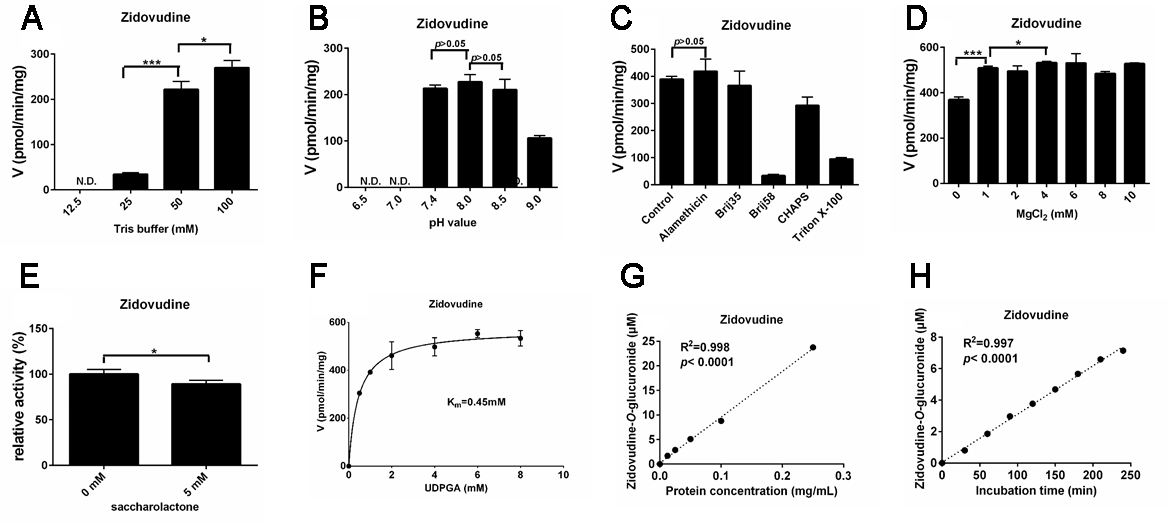


**Figure S5**


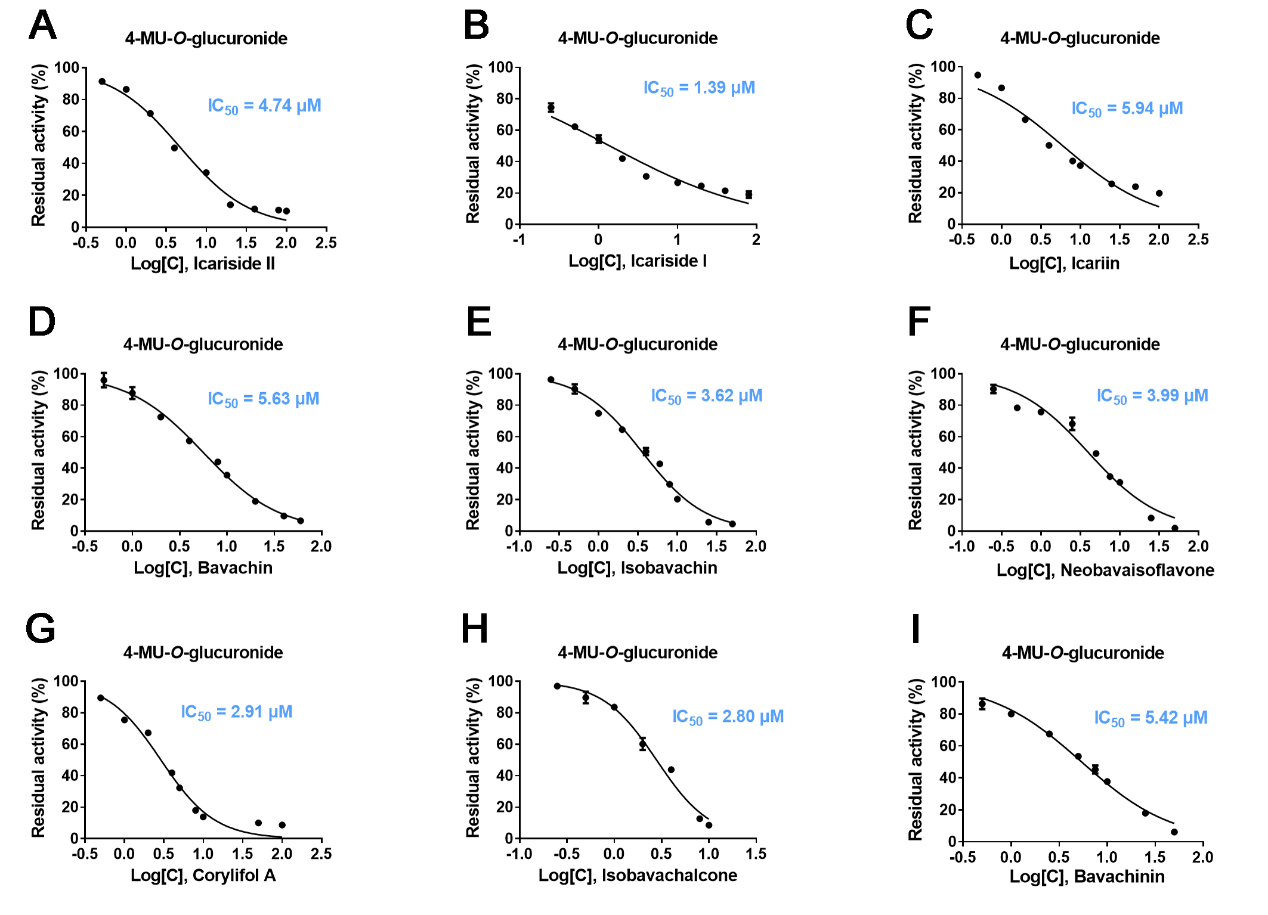


**Figure S6**


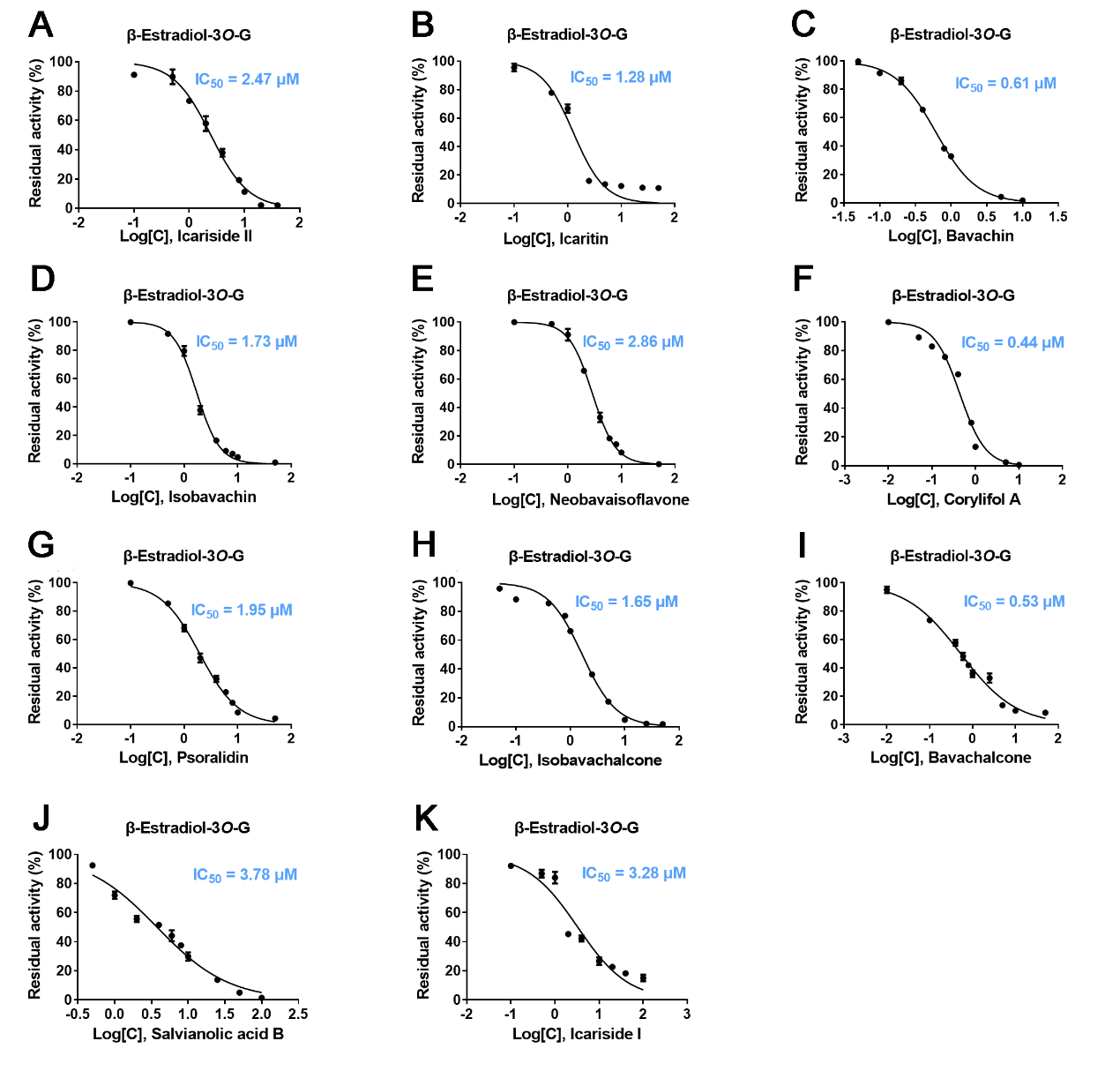


**Figure S7**


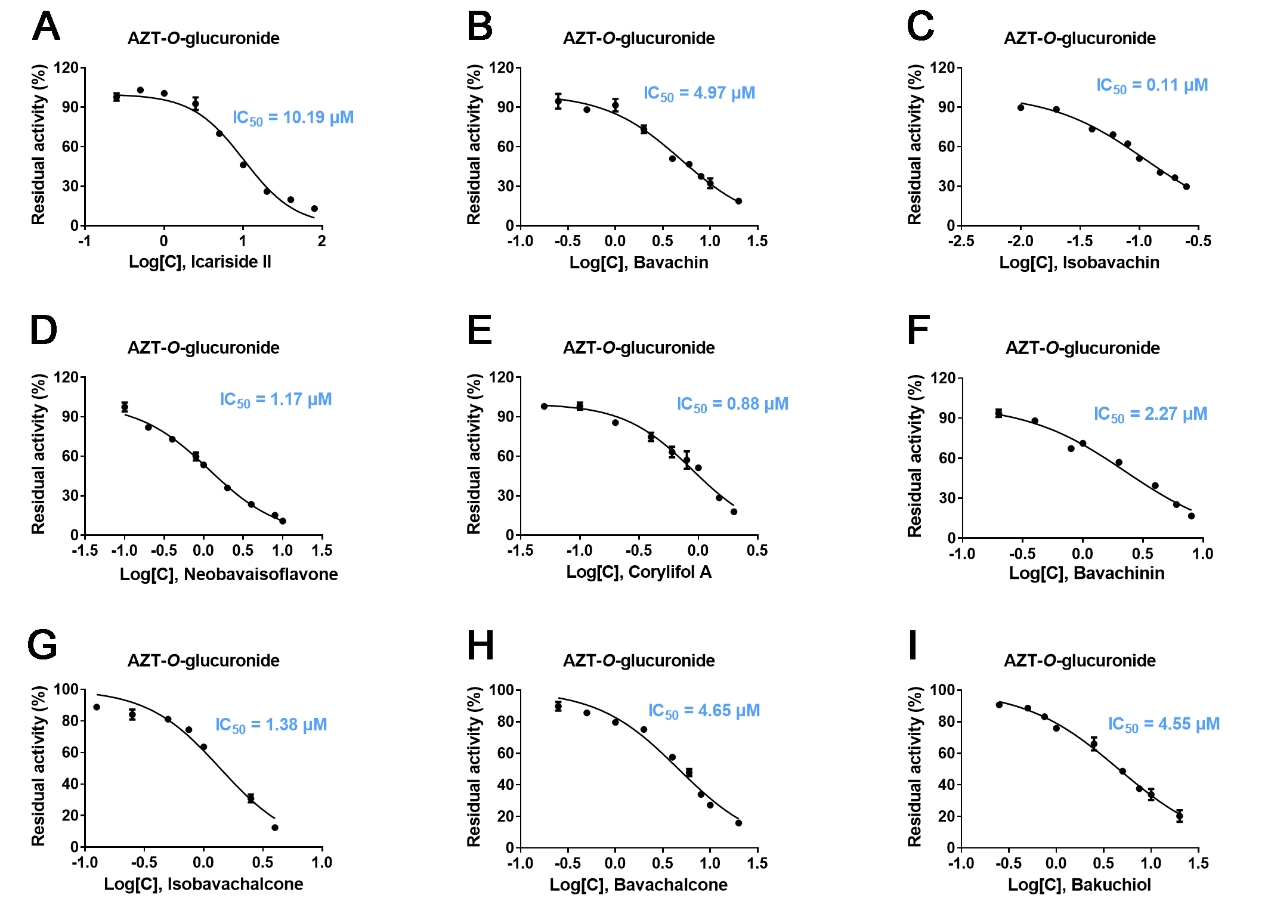


**Figure S8**


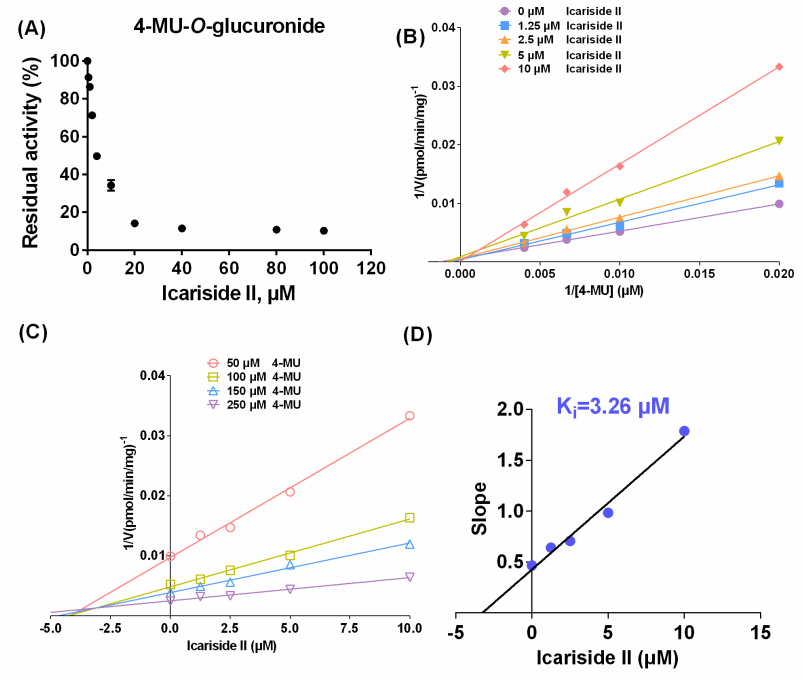


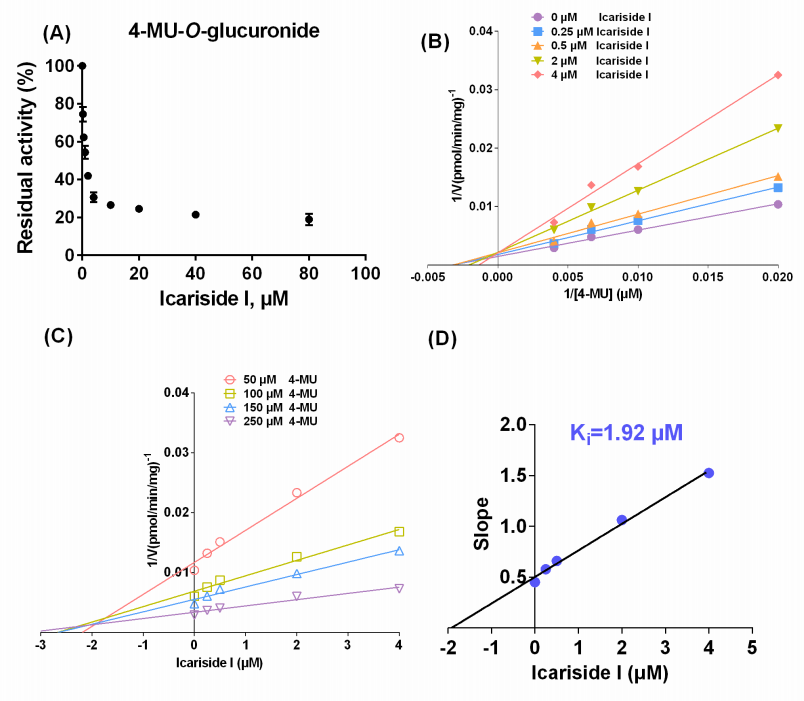


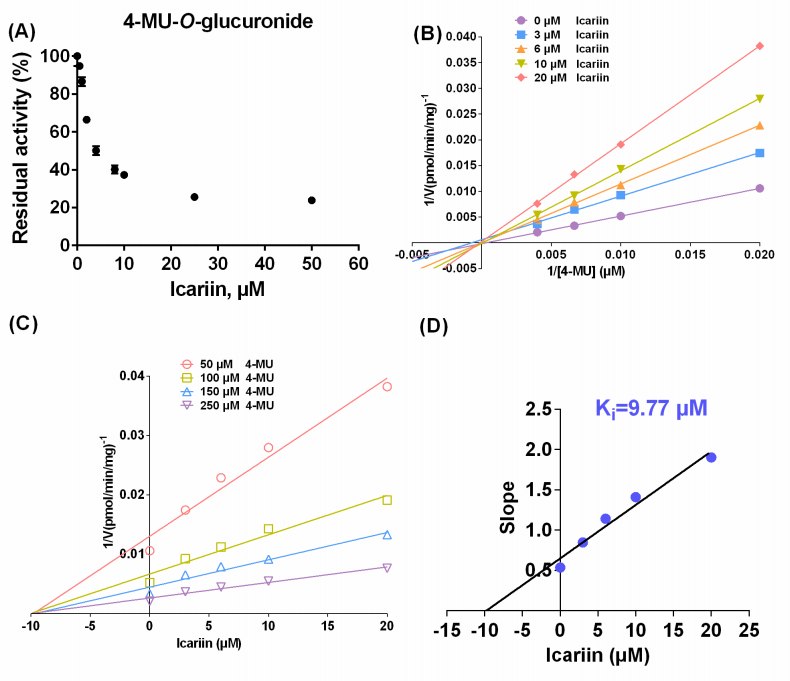


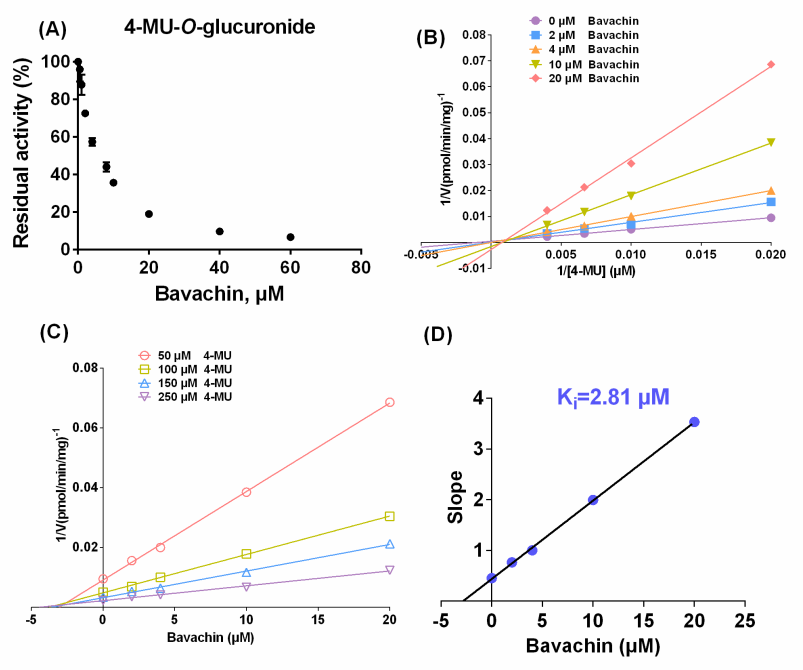


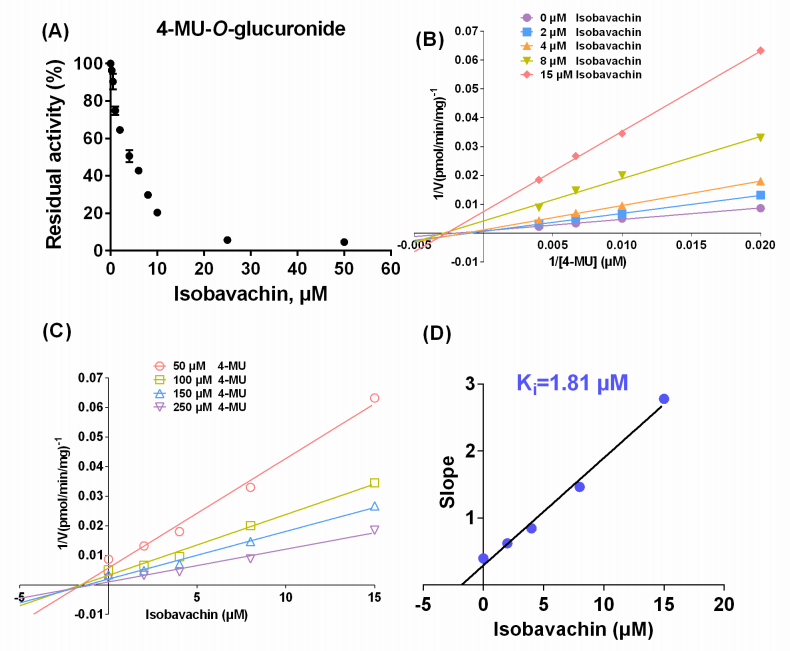


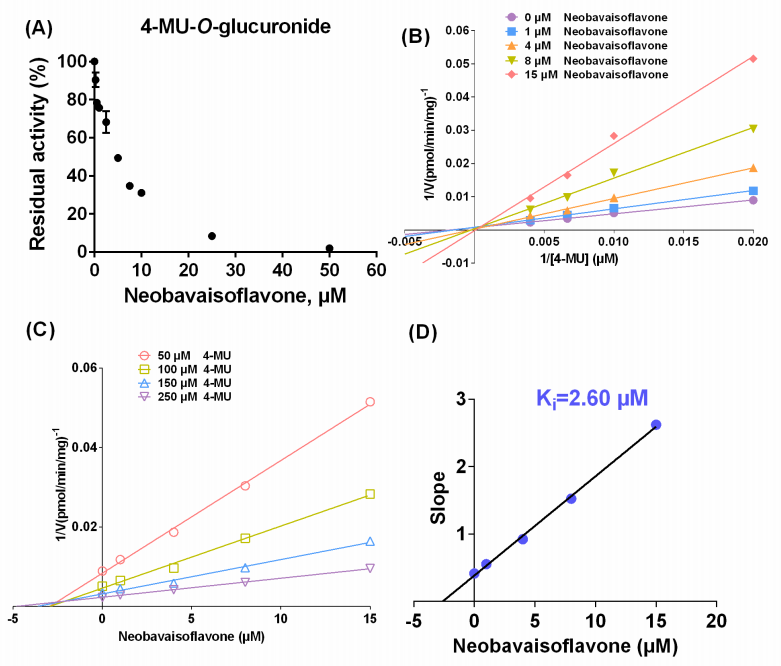


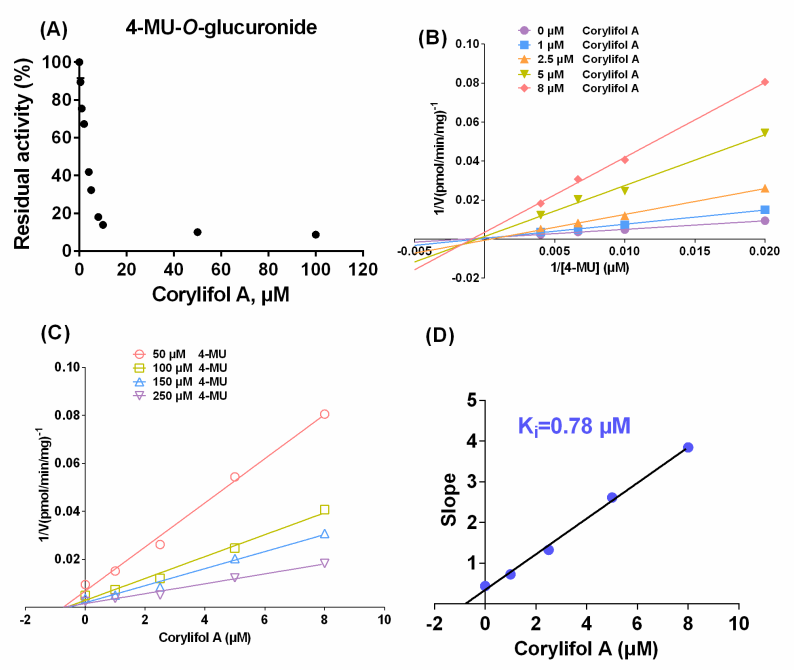


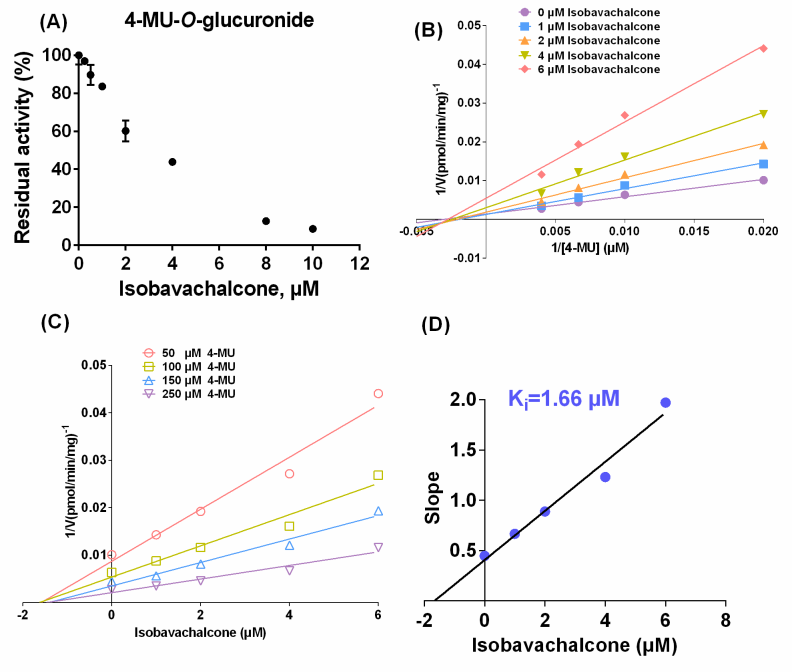


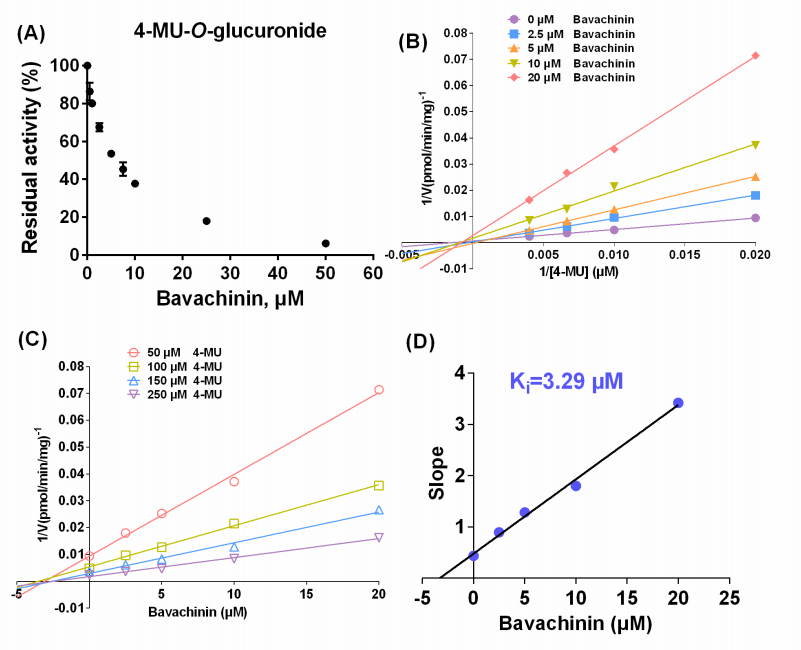


**Fig. S9**


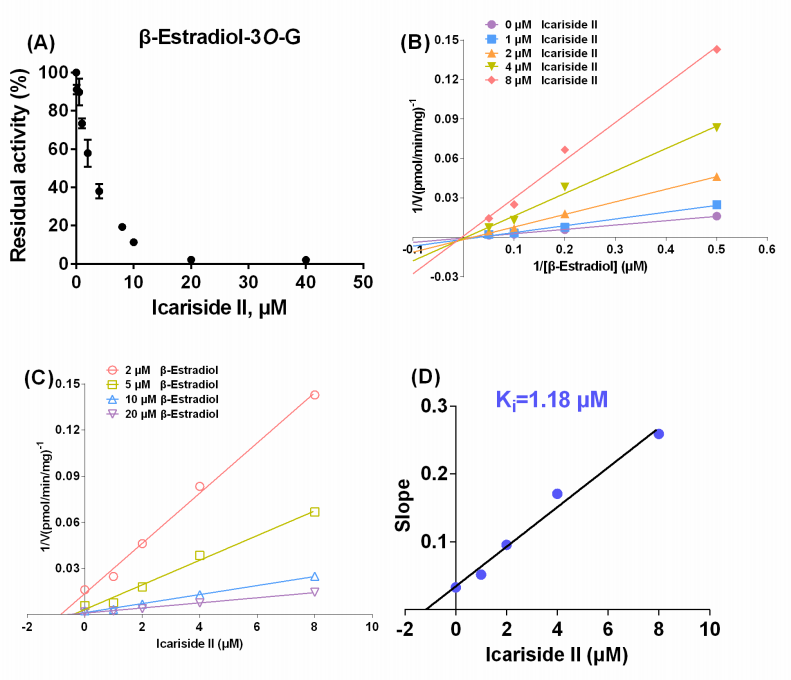


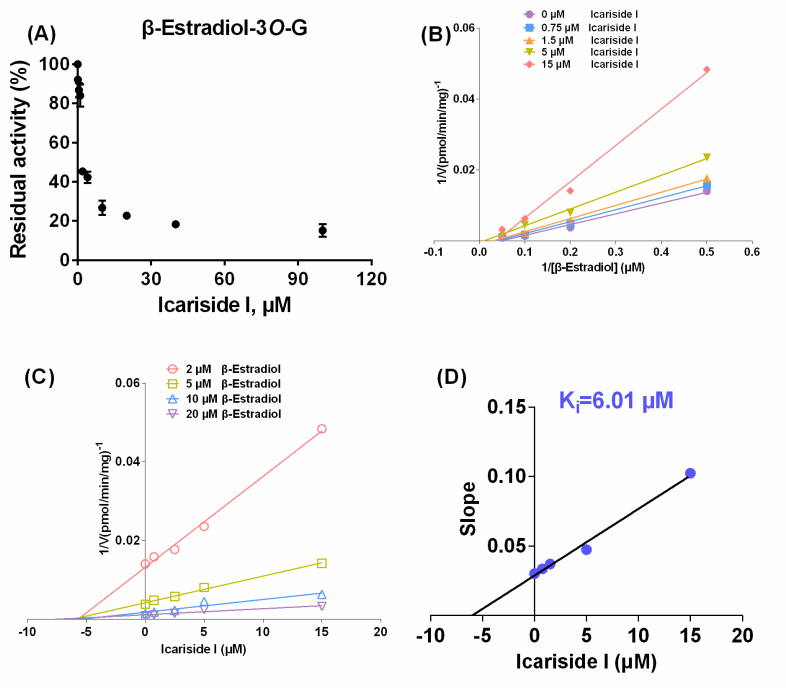


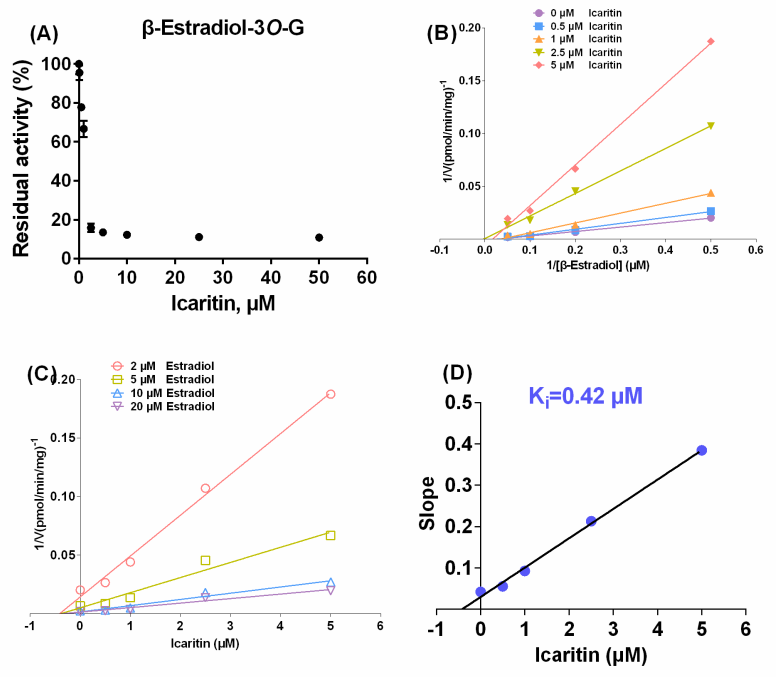


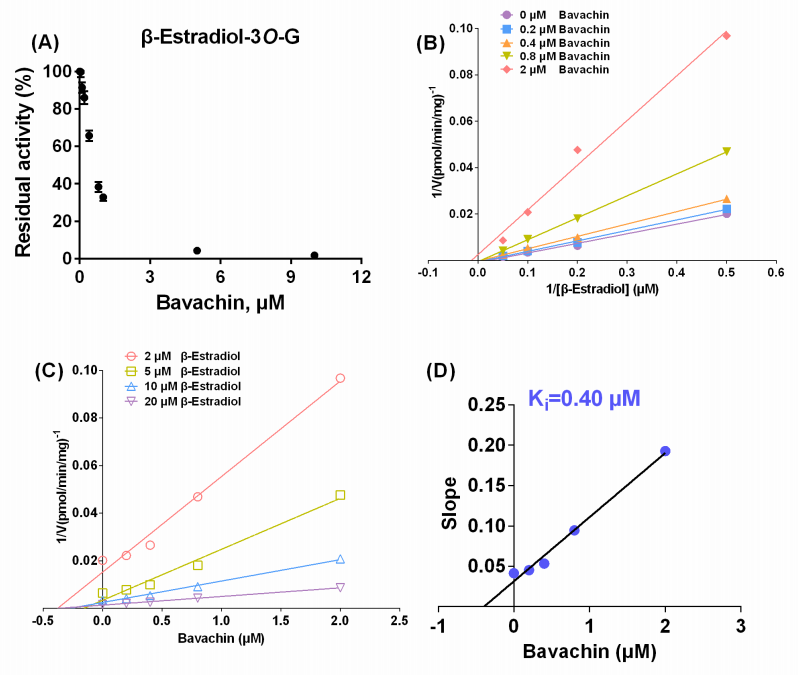


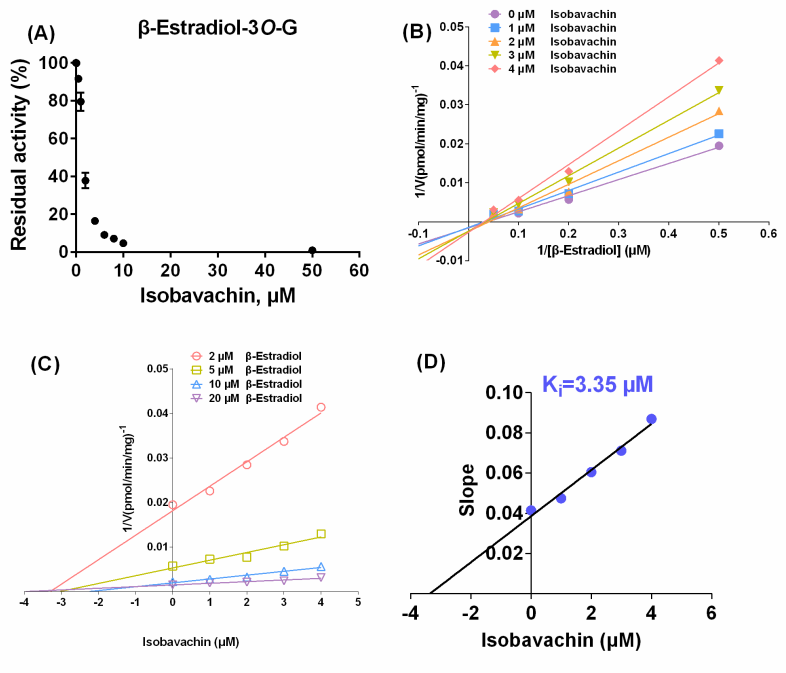


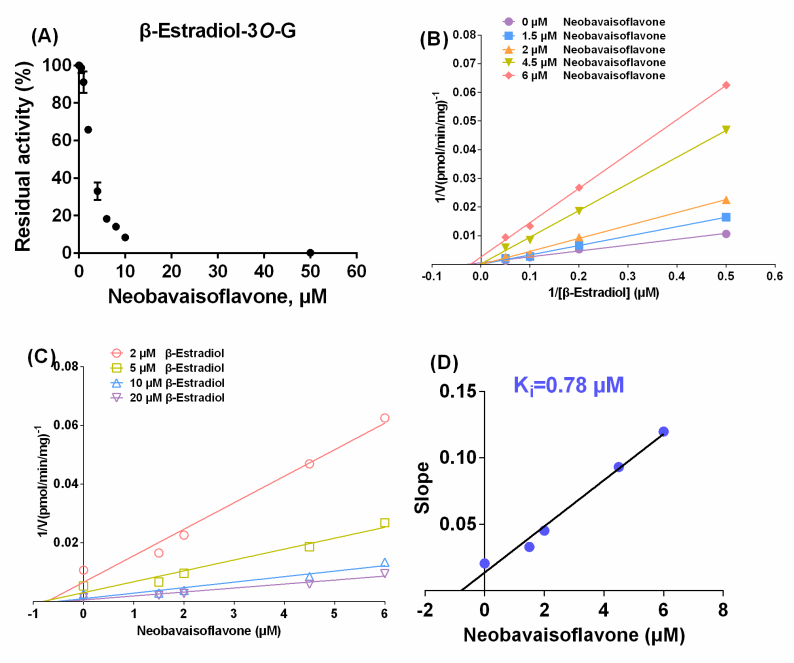


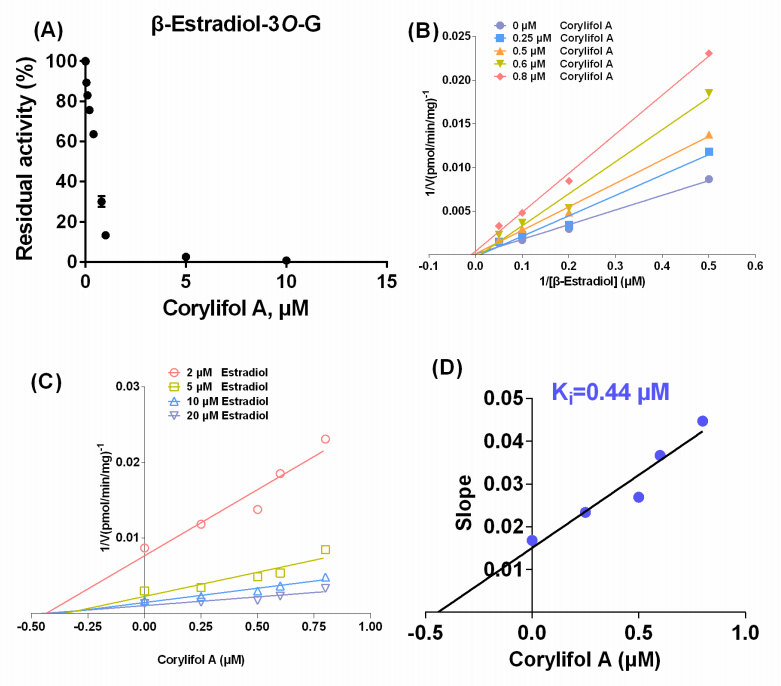


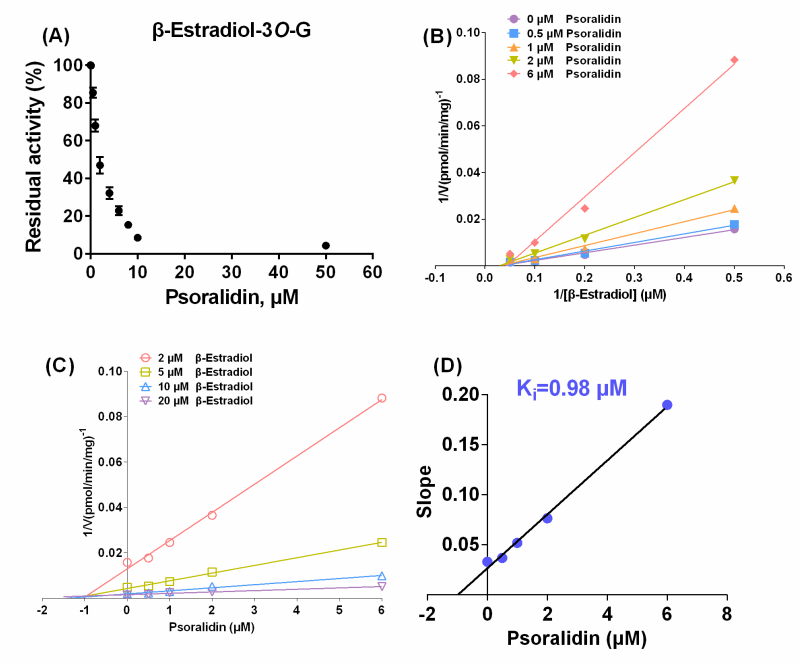


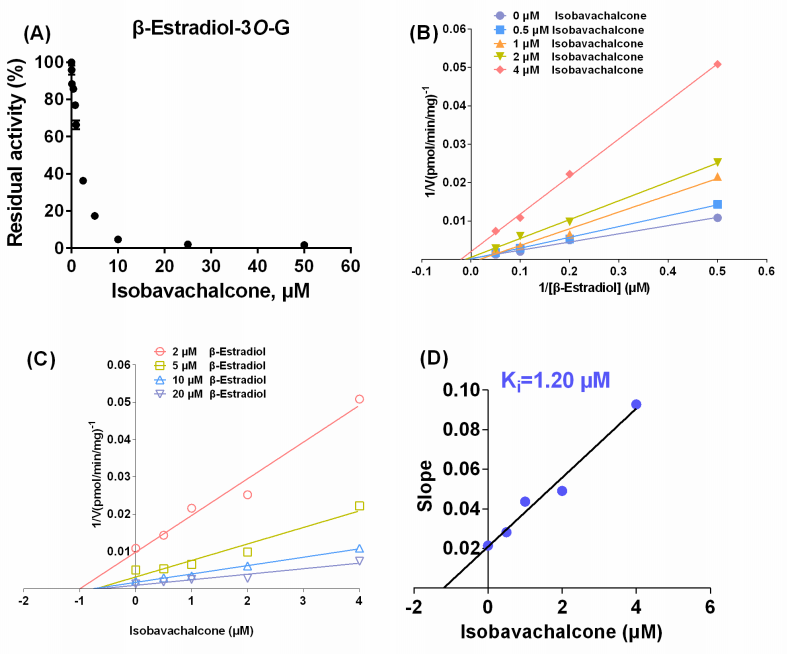


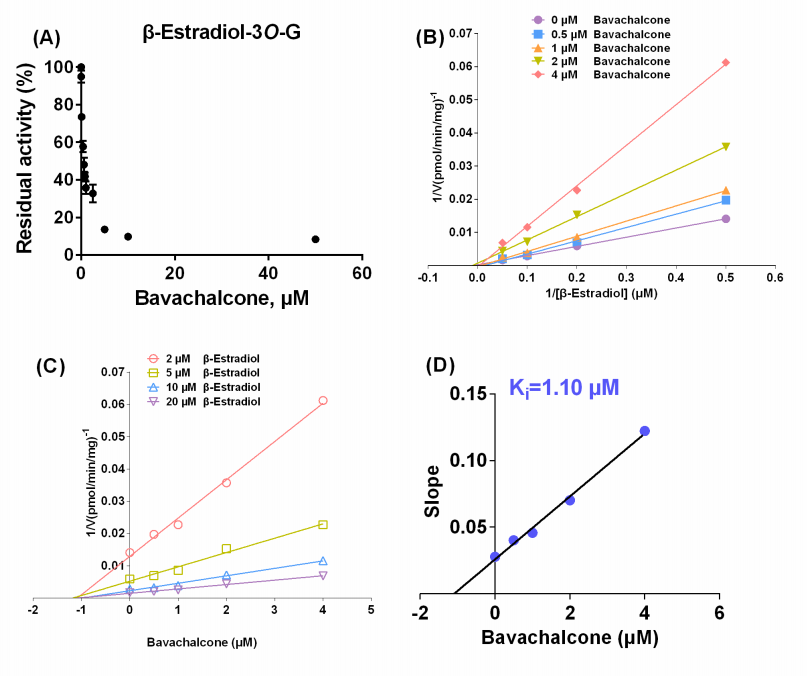


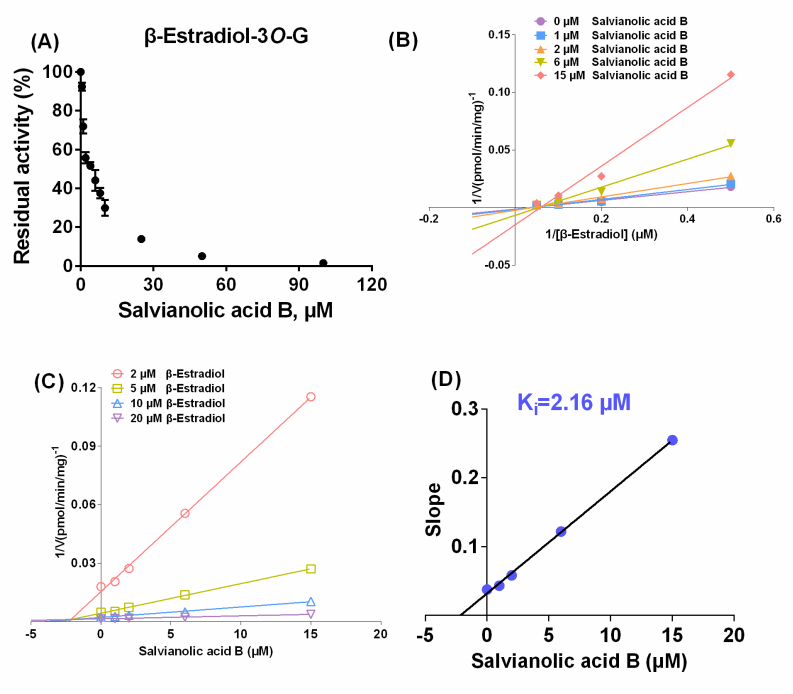


**Fig. S10**


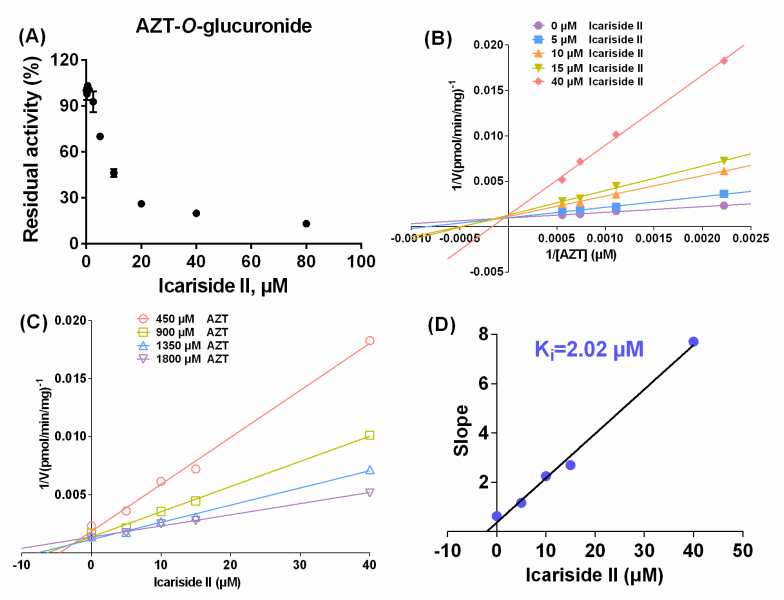


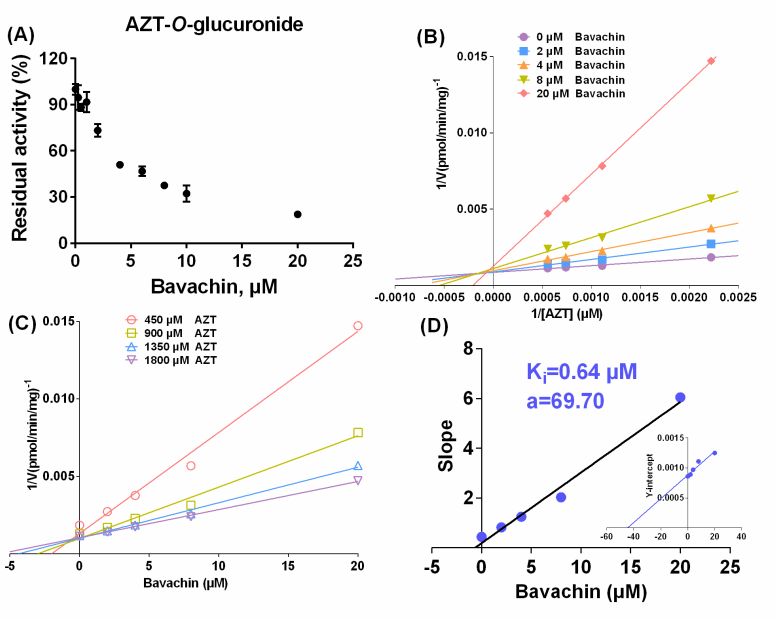


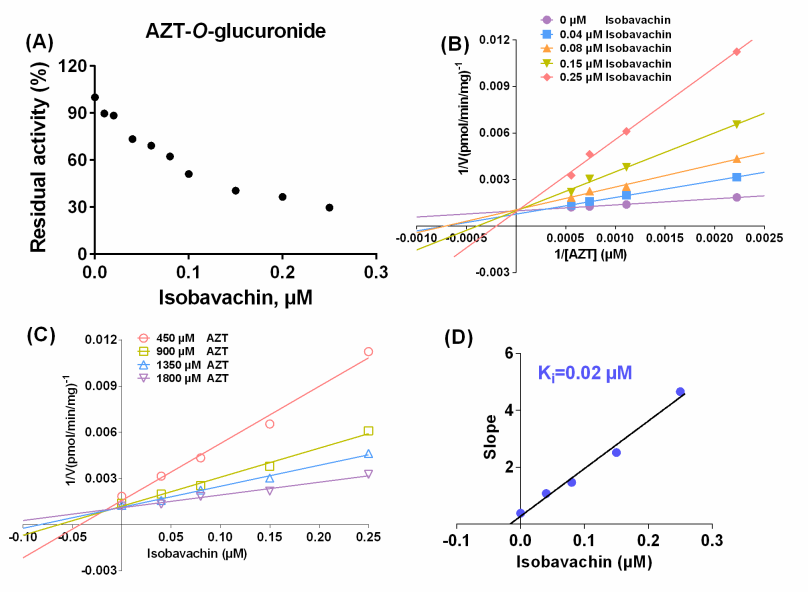


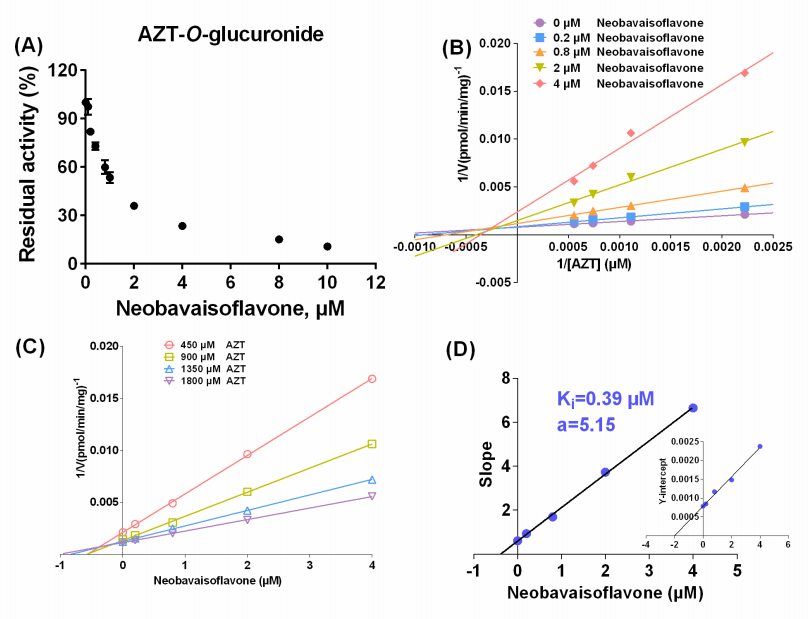


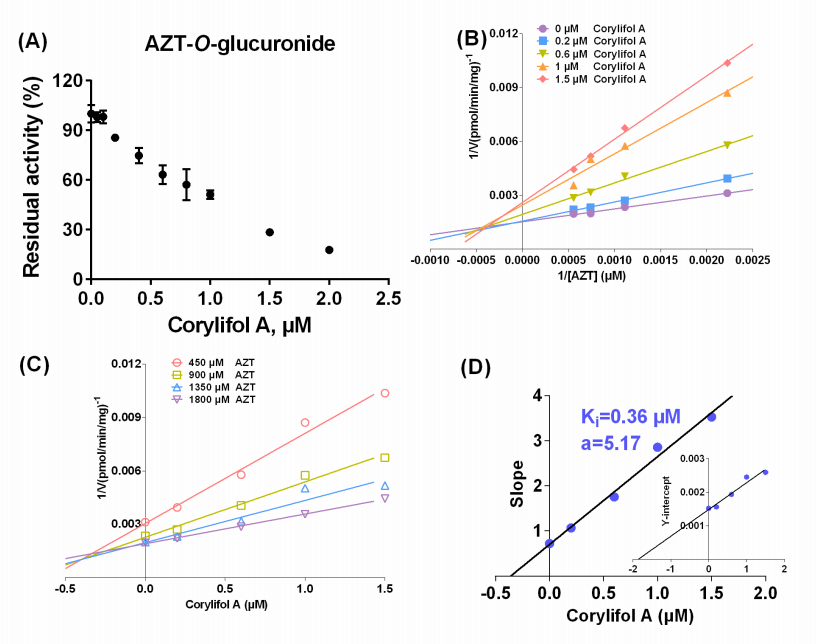


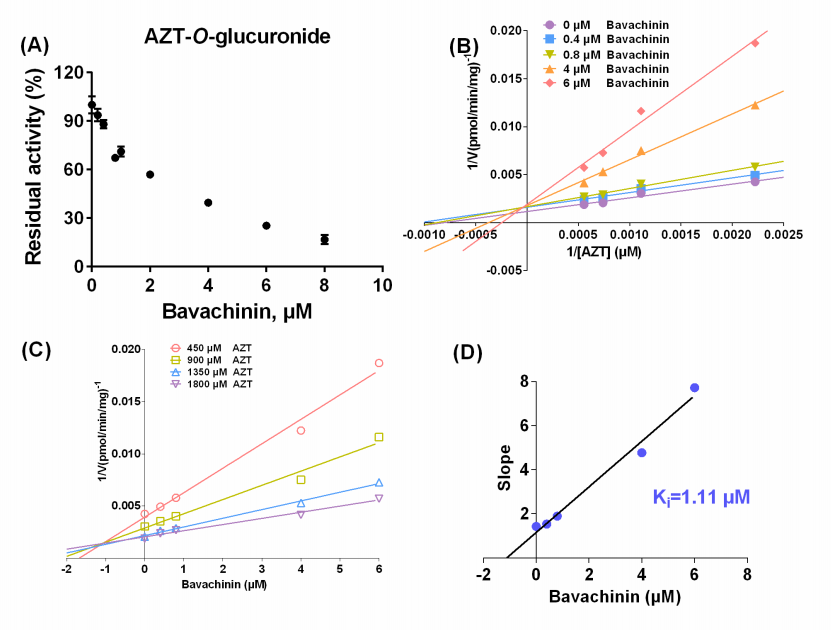


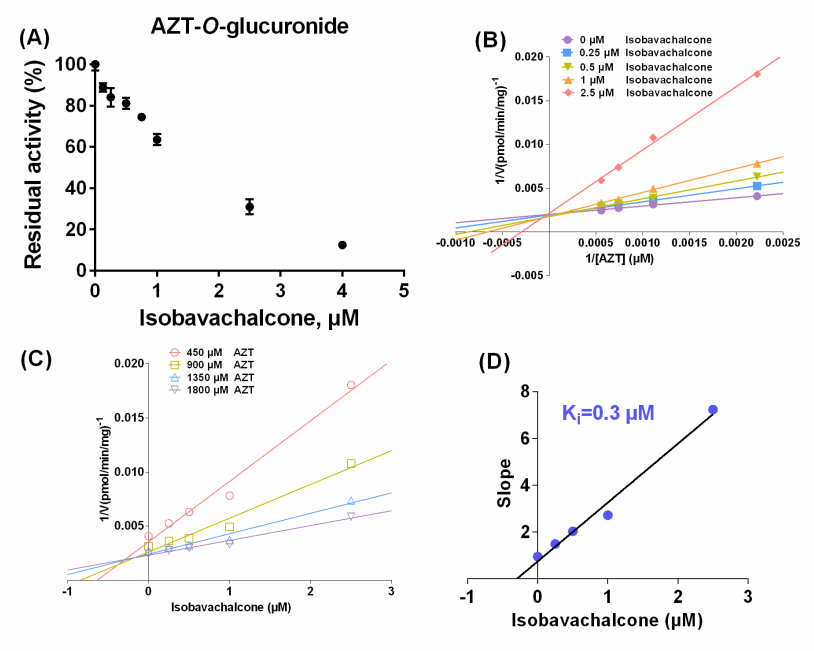


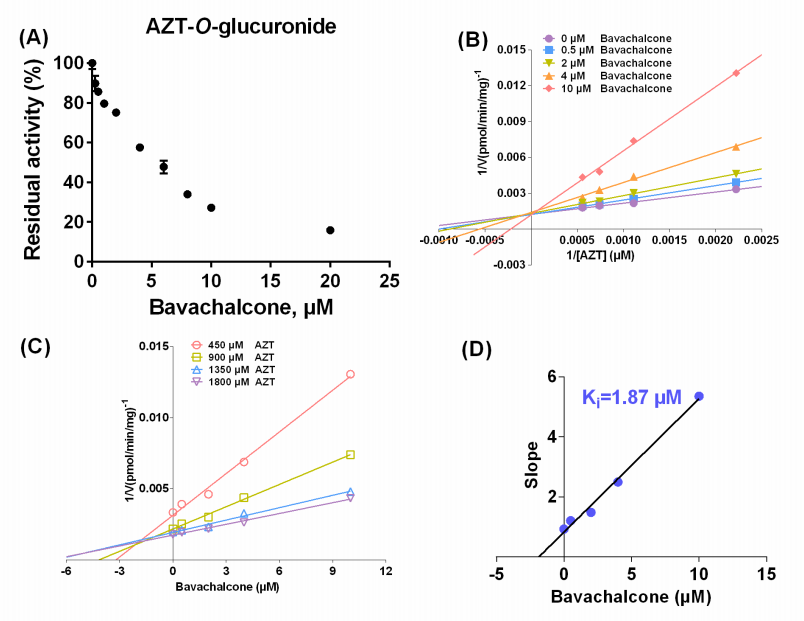


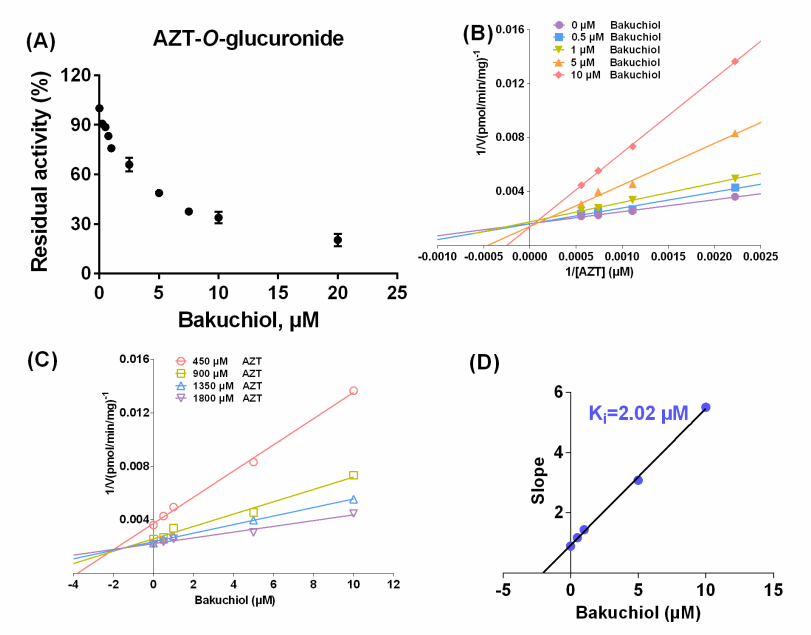


**Fig. S11**
